# Understanding polysulfide evolution in wine: insights from accelerated ageing and real-time cellaring in different packaging^☆^

**DOI:** 10.1016/j.fochx.2025.103447

**Published:** 2025-12-26

**Authors:** Yu Hou, Marlize Z. Bekker, Tracey E. Siebert, Gal Y. Kreitman, David W. Jeffery

**Affiliations:** a School of Agriculture, Food and Wine, and Waite Research Institute, The University of Adelaide, PMB 1, Glen Osmond, SA 5064, Australia; b School of Agriculture and Food Sustainability, The University of Queensland, St Lucia, QLD, 4072, Australia; c The Australian Wine Research Institute, PO Box 46, Glenside, SA 5064, Australia; d Gallo, Modesto, CA 95354, USA

**Keywords:** Volatile sulfur compounds, Disulfide, GC-SCD, Wine analysis, Stability, Redox reaction, Cysteine (PubChem CID: 124886), Cystine (PubChem CID: 67678), Dimethyl sulfide (PubChem CID: 1068), Glutathione (PubChem CID: 124886), Glutathione disulfide (PubChem CID: 65359), Hydrogen sulfide (PubChem CID: 402), Methanethiol (PubChem CID: 878), Sulfur dioxide (PubChem CID: 1119)

## Abstract

Volatile sulfur compounds (VSCs) and non-volatile biothiols play a crucial role in wine quality. Certain sulfhydryl compounds can react to form odourless polysulfides (RSS_n_SR′) that potentially contribute to the development of sulfur off-odours during winemaking and storage. This study investigated the evolution of sulfur species in Chardonnay and Shiraz wine during real-time and accelerated ageing in glass bottles with different closures and in aluminium cans. Moderate glutathione (GSH) accumulation was accompanied by disulfide formation, with GSH trisulfide (GS-S-SG), tetrasulfide (GS-S_2_-SG), and mixed cysteine-GSH tetrasulfide (Cys-S_2_-SG) displaying compound- and matrix-specific trends. Can packaging maintained higher amounts of VSCs, sulfur dioxide, and GSH, whereas closures with varying oxygen transmission had the most pronounced impact on glutathione disulfide (GS-SG) accumulation. Treatment with nitrogen gas or copper exhibited preservation effects under accelerated ageing. These findings highlight the role of wine packaging on polysulfide chemistry and clarify the effects of pre-bottling strategies for VSC management.

## Highlights

• Polysulfides assessed during real-time cellaring and accelerated ageing of wine.
• Correlations observed between polysulfides and volatile sulfur compounds.
• Packaging format (bottle or can) modulated sulfur compound temporal profiles.
• Adding glutathione and H_2_S altered sulfur compound evolution especially in red wine.
• Treatment with usual wine additives modified the concentration of sulfur compounds.

## 1 Introduction

Sulfur-containing compounds have long attracted attention in oenological research due to their pronounced impact on aroma and overall quality (Waterhouse et al., 2024g). These compounds include volatile and non-volatile forms. Volatile sulfur compounds (VSCs) can originate in the grape and are produced during fermentation or from transformations during ageing (Bonnaffoux et al., 2021; Kreitman et al., 2019; Smith et al., 2015; Waterhouse et al., 2024c). Hydrogen sulfide (H_2_S), methanethiol (MeSH), and dimethyl sulfide (DMS) are notable examples associated with sulfur-related (“reductive”) off aromas perceived as wine faults.

The non-volatile sulfur species include tripeptide glutathione (GSH), the main biothiol in grapes ranging from non-detected to 100 mg/L, and amino acid cysteine (Cys), typically below 20 mg/L (Kritzinger et al., 2013; Waterhouse, Sacks and Jeffery, 2024a, Waterhouse, Sacks and Jeffery, 2024c, Waterhouse, Sacks and Jeffery, 2024g). Other non-volatile species can develop in wine through binding with metals, polymerisation, or oxidation processes during and after winemaking. This leads to the presence of metal-sulfhydryl complexes (e.g., Cu(I)_x_-S_y_), thionates (e.g., S_4_O_6^2−^_), *S*-alkylthiosulfates (e.g., GS-SO_3^−^_), and disulfides (e.g., GS-SG), some of which are implicated in the reoccurrence of reductive aromas during wine storage (i.e., are latent sources of VSCs) (Kreitman et al., 2019; Müller et al., 2022; Tachtalidou et al., 2024; Waterhouse et al., 2024d).

Expanding on the concept of latent VSC sources, polysulfides (diorganopolysulfanes, RSS_n_SR′) in wine have emerged as a significant topic in wine chemistry (Bekker, Kreitman, et al., 2018; Kreitman et al., 2017). Involving redox phenomena, polysulfides are related to the metal-sulfhydryl pathway, a minor companion to the well-known metal-phenolic pathway associated with oxidation in wine (Waterhouse et al., 2024g). Their proposed role as a reservoir of VSCs has prompted investigations in wine related to their formation (Jastrzembski et al., 2017; Kreitman et al., 2017), occurrence (Dekker et al., 2020; Dekker et al., 2023; Dekker, Fedrizzi, van Leeuwen, Nardin, et al., 2022; Dekker, Fedrizzi, van Leeuwen, Roman, et al., 2022; Huang et al., 2023; Kreitman et al., 2019; Nardin et al., 2020; Pilkington et al., 2019; van Leeuwen et al., 2020), stability (Hou et al., 2025a), and degradation (Bekker, Kreitman, et al., 2018; Dekker et al., 2023; Hou et al., 2025a). Generally in the absence of commercially available authentic standards, studies have characterised GSH and Cys derived polysulfides using high-performance liquid chromatography (HPLC) with mass spectrometry (MS), with notable contributions by Kreitman et al. (2017), Jastrzembski et al. (2017), Bekker, Kreitman, et al. (2018), Pilkington et al. (2019), van Leeuwen et al. (2020), and Dekker et al. (2020). More recently, semi-quantitative approaches have been developed to determine polysulfide concentrations, with results expressed as equivalents of glutathione disulfide (GS-SG), trisulfide (GS-S-SG), or tetrasulfide (GS-S_2_-SG) (Hou, Bekker, Siebert and Jeffery, 2025a, Hou, Bekker, Siebert and Jeffery, 2025b).

Despite the advances in the understanding of polysulfides in wine, such as their identification and characterisation in *Saccharomyces cerevisiae* (Huang et al., 2023; Pilkington et al., 2019) and accumulation in white wine (Dekker, Fedrizzi, van Leeuwen, Nardin, et al., 2022; Dekker, Fedrizzi, van Leeuwen, Roman, et al., 2022), their behaviour and evolution under wine storage conditions remained largely unexplored, especially in a quantitative sense. Wine ageing regimes can significantly influence sulfur compound composition over time, not only by driving transformation of small molecular species into dimers or complex clusters, but also by promoting the gradual conversion of non-volatile precursors into aroma-active compounds (Goulioti et al., 2025; Jiménez-Lorenzo et al., 2022; Vela et al., 2017).

Packaging materials modulate wine ageing by regulating the oxygen ingress through closures (Lopes et al., 2006) and through potential surface interactions with alternative packages (Versari et al., 2023). Glass bottles and aluminium cans are generally considered low oxygen-permeable systems based on the detected oxygen transmission rates (OTRs) (Waterhouse et al., 2024f), whereas natural or synthetic corks generally allows greater oxygen entry than screw cap (SC) (Waterhouse et al., 2024e). Aluminium cans provide an almost hermetic seal but may also raise the risk of off-odour development from H_2_S, potentially resulting from aluminium corrosion with SO_2_ at wine pH when the polymeric lining is compromised (Montgomery et al., 2023; Sheehan et al., 2024; Versari et al., 2023).

According to the metal-sulfhydryl pathway (Waterhouse et al., 2024g), thiols can be transformed into polysulfides via copper-catalysed reactions, with oxygen and iron also playing a role (Kreitman et al., 2017). This suggested that redox-related factors warranted further exploration for their potential roles in polysulfide dynamics during wine storage. The aim of this study was therefore to investigate the evolution of di- and polysulfides under different packaging and ageing conditions. As commercially important representative varietals of Australian white and red wines, key sulfur species in a Chardonnay wine and a Shiraz wine were profiled by HPLC-MS/MS, and their correlations with certain VSCs, SO_2_, and GSH were examined to gain deeper insights into the chemistry of di- and polysulfides in wine. To simulate long-term storage, accelerated ageing trials were conducted by storing wine at elevated temperatures, in conjunction with 12-month natural ageing, allowing for an assessment of sulfur species stability under different packaging conditions.

## 2 Materials and methods

### 2.1 Chemicals, solutions, and wines

All chemicals were of analytical reagent or HPLC grade and solutions were prepared volumetrically (expressed as *v*/*v* unless otherwise indicated). VSC standards were prepared following Siebert et al. (2010) and Bekker, Kreitman, et al., 2018. Solutions of polysulfide standards were prepared according to Hou et al. (2025b). Sodium sulfide nonahydrate (Na_2_Sꞏ9H_2_O, 99.9 %), reduced l-glutathione (GSH, 98 %), *S*-hexylglutathione (hexyl-GSH, 98 %), *N*-ethylmaleimide (NEM, 98 %), and ethylenediaminetetraacetic acid (EDTA, 99 %) were obtained from Sigma-Aldrich (Castle Hill, NSW, Australia). Iron (III) sulfate pentahydrate (Fe_2_(SO_4_)_3_·5H_2_O, 97 %) was from Acros Organics (Scoresby, VIC, Australia), and copper (II) sulfate pentahydrate (CuSO_4_·5H_2_O, 99 %) was from Ajax Chemicals (Sydney, NSW, Australia). l-Ascorbic acid (99 %) was from Merck (Frenchs Forest, NSW, Australia) and potassium metabisulfite was from ChemSupply Australia (Gillman, SA, Australia). Water (18.2 MΩ/cm) was obtained from a Milli-Q purification system (Millipore, North Ryde, NSW, Australia). Model wine was prepared as described by Hou et al. (2025b).

Commercially-produced 2020 Shiraz (15.7 % v/v, McLaren Vale) and 2023 Chardonnay (13.5 % v/v, South Australia) wines were sourced from a local winery (Belvidere, SA, Australia). For each variety, there was an untreated control and a treatment wine spiked with 40 mg/L GSH and H_2_S (from Na_2_S·9H_2_O) to reach an initial level of 4 μg/L, yielding four wine types per packaging experiment: Sh and Ch denote the control Shiraz and Chardonnay wines, respectively, while Sh+ and Ch+ represent the spiked wines. The experimental design is presented in detail in Fig. A.1 (Appendix A).

### 2.2 Procedures for real-time ageing of wine

Screw caps (SCs) with different OTR (SC1: 0.002, SC2: 0.002-0.005, SC3: 0.005 mL/day/bottle) and 750 mL Bague Verre Stelvin (BVS) finish screw-top bottles were sourced from Interpack (Dudley Park, SA, Australia). Natural cork closures (44 × 24 mm) and 750 mL punted glass bottles were provided by the local winery that supplied the wines. Bottling was undertaken manually, with SCs sealed with a bench-top capping machine (JB Macmahon Pty Ltd, Forestville, SA, Australia) and corks inserted using a manual corker. Slimline 250 mL aluminium cans and associated canning facilities were provided by Affinity Labs (Adelaide, SA, Australia). Bottling and canning were undertaken in May 2024. Real-time ageing was conducted at 18–20 °C, with samples analysed at 3, 6, and 12 months.

### 2.3 Procedures for accelerated ageing of wine

The four wine types were aliquoted into 20 mL pre-scored ampoules (Sigma-Aldrich) and subjected to accelerated ageing at 37–38 °C in a laboratory oven for five weeks (Casassa et al., 2023). Experimental treatments comprised filling under nitrogen (N_2_) to minimise air exposure, filling under ambient air (control), and air with the addition of either SO_2_ (21 mg/L), ascorbic acid (AA, 40 mg/L), a combination of SO_2_ and AA (21 mg/L + 40 mg/L), iron (Fe, 4 mg/L), or copper (Cu, 4 mg/L). In addition, a set of the four wine types in aluminium cans was included in the accelerated ageing experiment under the same conditions as the ampoules (Fig. A.1, Appendix A), with both groups prepared in duplicate.

### 2.4 Chemical analyses

The concentration of SO_2_ in wine samples was determined using a ChemWell 2910 discrete analyser with Vintessential SO_2_ test kit purchased from Rowe Scientific (Lonsdale, SA, Australia). Brine-releasable VSCs were analysed using gas chromatography coupled with sulfur chemiluminescence detection (GC-SCD), following the method developed by Siebert et al. (2010). GSH and GS-SG were quantified using HPLC-MS/MS following GSH derivatisation with *N*-ethylmaleimide (NEM) using an adaptation of the method reported by Bekker et al. (2023), with modified eluents (each containing 0.5 % formic acid) and adjusted flow rate (0.4 mL/min). Polysulfide determination was conducted using solid-phase extraction (SPE) and analysis by HPLC-MS/MS according to the procedure of Hou et al. (2025b). All the measurements were performed in duplicate at every time point except initially for SO_2_ and H_2_S.

### 2.5 Data analysis

Results are presented as means ± SE. Statistical analyses were conducted using GraphPad Prism (version 10.1.0, GraphPad Software Inc., La Jolla, CA, USA). Repeated-measures two-way ANOVA with multiple comparisons was applied to analyse the effect of time and package formats, as well as the effect of time and co-spike treatment (GSH/H_2_S) of each analyte. Data were log_10_-transformed when necessary to meet assumptions of normality and homogeneity of variance. For concentrations below the limit of quantification (LOQ), data were replaced with a value of LOQ/2 prior to transformation (Ganser & Hewett, 2010). One-way ANOVA followed by Tukey's multiple comparison test was used to assess the changes under real-time cellaring and accelerated ageing within glass and can formats, and the effect of treatment (control vs co-spike treatments with GSH/H_2_S, ambient-air control vs N_2_-flushed or additive-supplemented ampoule treatment) under accelerated ageing. Normality was assessed (α = 0.05) and Spearman's correlation analysis was undertaken due to non-normal data distribution.

## 3 Results and discussion

### 3.1 Overview of experimental setup

To investigate the effects of white and red wine composition and packaging on sulfur chemistry during ageing, control group and GSH/H_2_S treated (co-spiked) wines were monitored over time (Fig. A.1, Appendix A). The level of GSH addition (Section 2.1) reflected a moderate concentration within the typical oenological range. A modest level of H_2_S was used to elevate the initial amount (less than 2.28 μg/L) for a more obvious effect, and to introduce additional sulfane sulfur source to facilitate polysulfide formation (Kreitman et al., 2017). Closures with distinct OTRs enabled the comparison of oxygen availability for bottled wine. Real-time ageing was conducted at ambient cellar conditions that were slightly higher than traditional cellaring temperatures (up to 15 °C). This provided a stable storage environment for wine preservation (Echave et al., 2021), while also allowing measurable ageing to occur. Although polysulfides degrade faster at elevated temperatures (Hou et al., 2025a), the chosen conditions were unlikely to promote reaction pathways that would not occur ordinarily.

### 3.2 Temporal accumulation of di- and polysulfides in wine under real-time ageing

#### 3.2.1 Predominance of GS-SG

**Fig. 1:**
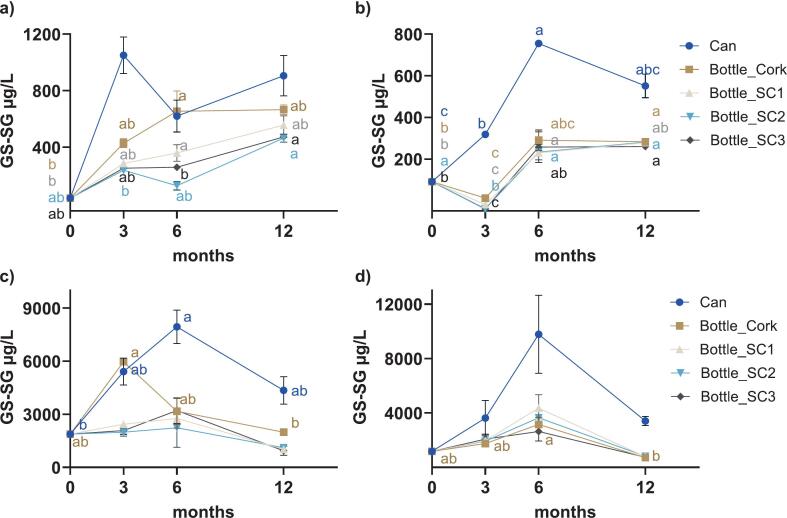
Temporal evolution of GS-SG concentrations (μg/L) during ageing in different packaging formats for a) Sh, b) Ch, c) Sh+, d) Ch+. Error bars represent standard error from duplicate measurements of each sample. Different lowercase letters in each panel (coloured to match the respective package according to the legend) designate significant differences over 12 months within the same package format (from two-way ANOVA on log_10_-transformed data, followed by Tukey's multiple comparison test, α = 0.05). SC, screw cap 1, 2, or 3; refer to Section 2.2 for details.

Real-time ageing revealed a gradual accumulation of Cys and GSH-derived di- and polysulfides according to periodic analysis over 12 months. However, the temporal patterns appeared to be influenced by multiple factors, including the specific sulfide species, wine type (red wine generally being higher), packaging format, and the addition of GSH and H_2_S. Among the quantified species, GS-SG consistently predominated, exhibiting the highest initial concentration (∼40 μg/L in Sh and 90 μg/L in Ch) and experiencing the most substantial increases during storage across all packaging types (average of 613 μg/L in Sh (Fig. 1a) and 331 μg/L in Ch (Fig. 1b) at 12 months). GS-SG rose into the mg/L range for wine spiked with GSH and H_2_S (Fig. 1c and d). In contrast, the concentrations of other disulfides—cystine (Cys-Cys), glutathione-MeSH disulfide (GS-SCH_3_), and cysteine-glutathione disulfide (Cys-SG)—were substantially lower, with the maximum accumulated levels not exceeding 22 μg/L (Fig. A.2 and A.3, Appendix A).

Two-way repeated measures ANOVA was conducted to investigate the effects of ageing time, package, and their interactions for di- and polysulfides (Table A.1, Appendix A). The concentration of GS-SG was significantly affected by time (*P* < 0.0001 for both Sh and Ch), package (*P* = 0.0005 and P < 0.0001, respectively) and by package × time (*P* = 0.0003 and P < 0.0001, respectively) over the 12-month ageing period (Table A.1). GS-SG concentrations fluctuated but increased over time in both Sh (Fig. 1a) and Ch (Fig. 1b), with post hoc mean comparisons on transformed data revealing significant differences between time points for certain packages, except for canned Sh. Canned wines exhibited markedly higher GS-SG accumulation (approximately double depending on time point, Fig. 1a and b) compared to their bottled counterparts, most prominently in canned Ch at 3 months vs cork (*P* = 0.0020). Both canned and cork-sealed Ch differed from all other SCs (*P* = 0.0211), potentially because the values of SC samples were below the LOQ. Additionally, canned Ch at 6 months differed from SC1 (*P* = 0.0001), and to a lesser extent, canned Sh at 3 months differed from SC3 (*P* = 0.0455, not all data shown). Among bottled wines, GS-SG levels in Sh were quite variable, beginning at 41 μg/L and rising up to 540 μg/L, whereas in Ch it increased from 90 μg/L to 275 μg/L, although bottle closure had no significant effect for either wine variety (data not shown).

Building on the pattern observed in control group, the samples supplemented with GSH and H_2_S at bottling demonstrated distinct GS-SG evolution (Fig. 1c and d). GS-SG in Sh+ was significantly influenced by time (P = 0.0020), package (*P* = 0.0027), and time × package interaction (*P* = 0.0150) (Table A.1, Appendix. A). Initially present at 1.9 mg/L, GS-SG peaked at 7.9 mg/L in can by 6 months, before ending at up to 4.4 mg/L (Fig. 1c), albeit significant differences were only observed for samples under cork and in can. Cork-sealed Sh+ wine exhibited a sharp rise by 3 months, reaching 6.0 mg/L and equivalent to canned wine at this stage. This was followed by a substantial decline to a final concentration of around 2 mg/L by 12 months. Bottles sealed with SCs behaved similarly to each other, without any significant change in GS-SG over the 12 months.

Two-way ANOVA revealed GS-SG concentrations in Ch+ were affected by the interaction of time × package (*P* = 0.0115) and by both individual factors of time (*P* < 0.0001) and package (*P* = 0.0002) (Table A.1, Appendix A). From an initial GS-SG concentration of 1.2 mg/L, bottled wines peaked at 6 months (with canned wine reaching up to 9.8 mg/L, but with large variability among the duplicates) before declining to below 1 mg/L at 12 months, although only cork revealed a significant change with time in Ch+ (Fig. 1d). The lack of significant differences between cork and SCs for Ch+ was consistent with the Ch results, whereas a distinct profile was observed for the canned sample at 12 months (*P* = 0.0486 vs cork, *P* = 0.0356 vs SC1, *P* ≥ 0.0525 for other SCs, not all data shown), again highlighting a unique effect of this package type. Two-way repeated measures ANOVA (Table A.2) confirmed the significant effect of the co-spiking treatment in both wine varieties when considering each package type (*P* ≤ 0.0066).

The other disulfides not only remained at low concentrations but also showed limited effects according to time × package interaction (*P* ≥ 0.3985) except for Cys-Cys in Sh (*P* = 0.0058). There was a significant simple effect for time in Sh (*P* ≤ 0.0035) and Ch (*P* ≤ 0.0014), whereas package remained non-significant (*P* ≥ 0.1025) except for GS-SCH_3_ in Sh (*P* = 0.0289) (Table A.1, Appendix. A). In detail, Cys-Cys in Sh declined significantly from 10.7 μg/L to 6.8 μg/L in bottles sealed with cork, SC1 and SC3 (Fig. A.2a, Appendix A). In Ch, Cys-Cys displayed a slight accumulation from 2.1 μg/L to 3.1 μg/L over ageing, with significant differences in canned and cork-sealed samples (Fig. A.2b). GS-SCH_3_ increased up to two-fold in Sh (Fig. A.2c) and Ch (Fig. A.2d), with a greater number of statistically significant comparisons observed for Sh (can, cork, and SC1) than Ch (SC3). Cys-SG displayed a fluctuating trend, with a significant difference over time observed under cork and SC1 in Sh (Fig. A.2e) and sizeable accumulation in Ch (Fig. A.2f) across all packages, from 2.3 μg/L to 11.8 μg/L and above. No significant differences in these three disulfides were observed among the different package types for either wine in contrast to GS-SG, potentially because higher initial GSH concentrations may facilitate the formation of GS-SG.

Upon addition of GSH and H_2_S, a significant effect of time (*P* ≤ 0.0179) was consistently observed for Cys-Cys, GS-SCH_3_, and Cys-SG in co-spiked wines (Table A.1, Appendix A). In addition, Cys-SG in Sh+ was affected by package (*P* = 0.0303) and time × package interaction (*P* = 0.0079), with Ch+ also revealing an interaction of time × package (*P* = 0.0326). In Sh+, Cys-Cys showed a decrease from 10.1 μg/L to 6.7 μg/L by 6 months, with recovery to near initial levels afterwards. Except for the canned wine, which did not change over time, significant differences were detected in Sh+ sealed with cork, SC2, and SC3 (Fig. A.3a, Appendix A). In Ch+, Cys-Cys increased from 2.1 μg/L to approximately 3 μg/L during ageing (Fig. A.3b), with significant changes noted for wine in can (3.1 μg/L) and bottle with cork closure (3.3 μg/L). Notably, GS-SCH_3_ increased substantially in Sh+ from 9.6 μg/L up to 43.0–76.2 μg/L, with significant differences observed across all packages (Fig. A.3c). In Ch+, the concentration rose from 2.0 μg/L to an average across package types of 4.4 μg/L, with significant changes observed for SC2 and SC3 (Fig. A.3d). Cys-SG differed among the two co-spiked wine varieties. There was an increasing trend from an initial level of 11.9 μg/L to 48.8 μg/L at 12 months in canned Sh+, albeit without a significant difference between the time points (*P* ≥ 0.0587) (Fig. A.3e). In Ch+, Cys-SG increased significantly from 2.3 μg/L to 17.8–28.9 μg/L at 12 months for all package formats (Fig. A.3f).

Package-related differences were detected only in Sh+, including can vs cork for Cys-Cys at 3 months (*P* = 0.0312) and GS-SCH_3_ at 12 months (*P* = 0.0300), and cork-sealed Sh+ for Cys-SG at 3 months vs SC2 (*P* = 0.0369) and SC3 (*P* = 0.0359, not all data shown). Two-way ANOVA for GSH/H_2_S co-spiking (Table A.2, Appendix A) indicated that the treatment generally affected Cys-Cys, GS-SCH_3_, and Cys-SG across packaging formats in Shiraz variety, except for Cys-Cys under SCs. In Chardonnay wine, there were no significant effects of the co-spiking treatment apart from Cys-SG for wine in can and in bottle under cork and SC2 (Table A.2). This implies that addition of GSH can facilitate the formation of additional GSH-related compounds, potentially as a function of the pool of available sulfhydryls (i.e., Cys, MeSH) that could differ by wine type (red vs white) and packaging. Supplementation with GSH, either before fermentation (Schmidt et al., 2020; Xu et al., 2019) or afterwards (Fracassetti et al., 2013) has previously been shown to increase the concentration of GS-SG, and the same could occur for mixed disulfides as seen in the present work.

#### 3.2.2 Factors affecting disulfide accumulation

These differences in disulfide accumulation can be related to oxygen availability as a function of packaging material and red versus white wine matrix. Studies have reported a continuous decrease in GSH concentration during winemaking and ageing. Its oxidised dimer GS-SG forms along with the depletion of GSH (Kritzinger et al., 2013). The pronounced accumulation of GS-SG observed in Shiraz samples sealed with cork (Fig. 1a and c) may be attributed to the higher OTR of natural cork compared to SCs (Suhas et al., 2025). Although GS-SG also accumulated in Chardonnay (Fig. 1b and d), there were no significant differences among the closure types. The contrast may reflect a distinct oxidative capacity of the matrix. Red wines are rich in antioxidant polyphenols and typically require lower SO_2_ dosage than white wines (e.g., total SO_2_ of 87.5 vs 159 mg/L, Table 1). Oxidation of polyphenols may help consume transmitted oxygen, resulting in similar profiles among SC groups in the red wine, whereas continual oxygen ingress through cork may have promoted GS-SG formation over time. In Ch, antioxidant protection mainly derives from SO_2_ and sufficient SO_2_ likely scavenged the oxidation products arising due to ingress of oxygen under cork, limiting the effect of closure type. The other two GSH-derived disulfides (Fig. A.2c–f, Appendix A) also showed significant temporal change.

**Table 1:** Concentrations of brine-releasable H_2_S, total SO_2_, and GSH in control and co-spiked (GSH/H_2_S) Shiraz and Chardonnay wines in different packages monitored during ageing.

		SO_2_ (μg/L)				H_2_S (μg/L)				GSH (mg/L)			
		0 m	3 m	6 m	12 m	0 m	3 m	6 m	12 m	0 m	3 m	6 m	12 m
Sh	Can	87.5a	30.5 ± 4.5b	29.8 ± 2.7ab	18.3 ± 2.0b	3.8a	2.3 ± 0.0b	< 0.2c	1.1 ± 0.1bc	0.0 ± 0.0b	0.1 ± 0.0ab	0.1 ± 0.0ab	0.3 ± 0.0a
	Cork	87.5a	59.1 ± 4.3ab	51.5 ± 0.2ab	38.0 ± 3.2b	2.8a	2.4 ± 0.0ab	1.5 ± 0.1c	0.9 ± 0.1bc	0.0 ± 0.0b	0.1 ± 0.0ab	0.1 ± 0.1ab	0.3 ± 0.0a
	SC1	87.5a	58.4 ± 3.1a	51.2 ± 2.0ab	36.9 ± 2.8b	2.8	2.4 ± 0.0	0.8 ± 1.1	1.3 ± 0.5	0.0 ± 0.0	0.1 ± 0.0	0.1 ± 0.0	0.3 ± 0.1
	SC2	87.5ab	58.5 ± 1.1a	56.2 ± 1.4ab	38.9 ± 0.4b	2.8a	2.5 ± 0.3ab	0.8 ± 1.1ab	0.9 ± 0.1b	0.0 ± 0.0	0.1 ± 0.0	0.1 ± 0.0	0.3 ± 0.0
	SC3	87.5ab	55.6 ± 1.6a	47.2 ± 3.9ab	36.5 ± 1.5b	2.8	2.5 ± 0.2	0.7 ± 1.1	1.4 ± 0.5	0.0 ± 0.0b	0.1 ± 0.0b	0.1 ± 0.1ab	0.3 ± 0.0a
Sh+	Can	87.5a	28.7 ± 2.9ab	15.5 ± 8.8b	5.9 ± 6.8ab	3.8a	2.7 ± 0.4ab	1.5 ± 0.2a	1.1 ± 0.2b	55.7 ± 7.9	2.6 ± 0.5	1.3 ± 0.63	0.8 ± 0.2
	Cork	87.5a	62.2 ± 0.9ab	53.1 ± 7.7b	36.9 ± 1.0ab	2.8a	3.0 ± 0.5ab	1.8 ± 0.1ab	1.4 ± 0.1b	55.7 ± 7.9ab	24.7 ± 1.4a	6.7 ± 1.8b	2.4 ± 0.1b
	SC1	87.5	57.3 ± 1.2	51.6 ± 4.0	35.9 ± 0.3	2.8a	2.4 ± 0.1b	1.6 ± 0.1b	1.1 ± 0.2ab	55.7 ± 7.9ab	9.7 ± 0.6a	4.7 ± 2.0ab	1.1 ± 0.3b
	SC2	87.5	56.7 ± 0.2	57.6 ± 2.0	35.2 ± 4.2	2.8a	2.4 ± 0.1b	1.6 ± 0.0c	1.1 ± 0.2abc	55.7 ± 7.9	7.5 ± 2.0	4.4 ± 2.8	1.2 ± 0.2
	SC3	87.5a	59.4 ± 0.3ab	47.7 ± 1.8ab	36.4 ± 3.9b	2.8a	2.4 ± 0.0b	1.6 ± 0.1c	1.3 ± 0.2bc	55.7 ± 7.9	8.6 ± 2.4	5.1 ± 1.9	1.0 ± 0.4
Ch	Can	159	142.0 ± 0.5	134.7 ± 1.0	131.1 ± 3.2	2.3a	2.9 ± 0.6ab	2.3 ± 0.1b	2.2 ± 0.3ab	0.1 ± 0.0	0.9 ± 0.1	0.1 ± 0.0	0.4 ± 0.0
	Cork	159	143.1 ± 3.9	136.3 ± 2.2	135.8 ± 1.5	2.3	3.2 ± 1.0	1.9 ± 0.2	1.5 ± 0.6	0.1 ± 0.0	1.0 ± 0.3	0.4 ± 0.0	0.4 ± 0.0
	SC1	159	141.3 ± 1.4	137.5 ± 2.1	133.1 ± 1.2	2.3	2.9 ± 0.4	2.1 ± 0.2	3.2 ± 0.1	0.1 ± 0.0	0.9 ± 0.5	0.3 ± 0.1	0.5 ± 0.1
	SC2	159	140.9 ± 1.0	135.5 ± 0.9	132.4 ± 1.9	2.3b	2.5 ± 0.1a	1.7 ± 0.1a	1.9 ± 0.1a	0.1 ± 0.0	0.9 ± 0.6	0.5 ± 0.4	0.4 ± 0.1
	SC3	159	141.2 ± 1.4	134.5 ± 0.1	134.3 ± 3.0	2.3b	2.5 ± 0.0a	2.1 ± 0.5ab	4.1 ± 0.3ab	0.1 ± 0.0	0.4 ± 0.6	0.4 ± 0.0	0.5 ± 0.0
Ch+	Can	159	147.3 ± 2.8	139.3 ± 1.2	134.6 ± 0.3	4.0	3.0 ± 0.5	3.9 ± 0.3	7.3 ± 2.1	54.4 ± 3.4a	21.8 ± 2.3b	12.7 ± 4.9ab	3.1 ± 0.1ab
	Cork	159a	141.4 ± 11.3ab	138.0 ± 3.8b	139.2 ± 0.4ab	3.9a	2.6 ± 0.0b	1.9 ± 0.1b	1.3 ± 0.2b	54.4 ± 3.4a	20.8 ± 2.7b	13.9 ± 6.0ab	2.8 ± 0.5b
	SC1	159ab	142.3 ± 1.2a	135.9 ± 3.4ab	137.6 ± 1.2b	3.9	2.8 ± 0.3	2.8 ± 0.1	4.6 ± 0.7	54.4 ± 3.4ab	21.5 ± 0.6a	11.9 ± 5.6ab	3.0 ± 0.0b
	SC2	159a	145.5 ± 3.4ab	138.3 ± 2.9b	132.9 ± 0.9ab	3.9a	2.7 ± 0.0b	1.9 ± 0.1b	1.6 ± 0.3ab	54.4 ± 3.4a	19.9 ± 0.4a	13.9 ± 5.4ab	2.7 ± 0.7b
	SC3	159ab	144.1 ± 1.6a	137.4 ± 1.3b	136.0 ± 1.5ab	3.0a	2.6 ± 0.0b	2.6 ± 1.1ab	7.6 ± 2.6ab	54.4 ± 3.4a	20.3 ± 1.7ab	16.0 ± 7.3ab	2.6 ± 0.5b

Values are expressed as mean ± SE (*n* = 2) at 3, 6, and 12 months whereas initial concentrations of H_2_S and SO_2_ at 0 months were determined without replication. Different lowercase letters designate significant differences over 12 months (time as fixed factor) within the same package format (from two-way repeated measures ANOVA followed by Tukey's multiple comparison test, α = 0.05).

GS-SG appeared to be the most favoured product under gradual oxygen exposure, both in terms of the concentration and accumulation trends over the 12-month ageing period in control wines (Fig. 1a and b). In addition to the formation, the concentration of disulfides in wine may be influenced by their subsequent consumption or transformation in a wine matrix. *S*-sulfonated GSH (i.e., GSSO_3^−^_) rather than GS-SG has been proposed as the predominant product of GSH after ageing bottled wine for 3 months (Dienes-Nagy et al., 2022). The progress of sulfonation reactions with thiols was found to be time-dependent and occurred regardless of oxidative environment (Tachtalidou et al., 2024). A high GS-SG/SO_2_ or GSH/SO_2_ ratio can significantly promote *S*-sulfonate formation by facilitating the cleavage (sulfitolysis) of the disulfide bond (Arapitsas et al., 2016). These findings help rationalise why the GSH and H_2_S supplemented wines behaved differently (Fig. 1c and d), whereby the added GSH facilitated the formation of additional GS-SG, which has the potential to react with SO_2_ and produce the sulfonate adduct of GSH (Arapitsas et al., 2016; Dienes-Nagy et al., 2022). This latter aspect could account for the fluctuation in GS-SG levels, with the establishment of equilibria among sulfur species over the later stages of ageing in both co-spiked groups.

Given the low oxygen ingress in canned wine, the formation of GS-SG in these samples was unlikely to be driven solely by oxygen. On the contrary, once the limited oxygen from canning was consumed, the resulting anoxic conditions likely promoted the release of free sulfhydryls from precursors (Ferreira et al., 2014; Franco-Luesma & Ferreira, 2016). Although aluminium cans are typically lined with a micron-scale polymer to prevent the direct contact of wine and metal, SO_2_ in wine can degrade the liner, leading to corrosion of the can (Montgomery et al., 2023; Waterhouse et al., 2024f). Elevated H_2_S release has been reported in canned wines, with aluminium concentrations also accumulating over time (Allison et al., 2022; Scrimgeour et al., 2020; Versari et al., 2023). Compared to bottled wines, aluminium in cans may also consume SO_2_ (Versari et al., 2023), which could have contributed to the consistently lower levels in canned Sh (Table 1). As a result, the limited availability of SO_2_ may have restricted the conversion of GS-SG into sulfonated products. In Ch, SO_2_ remained relatively high, with minor difference across all packaging types during ageing (Table 1). This suggests that GS-SG preservation in canned Ch was not due to the limited sulfitolysis by SO_2_, but rather attributed to the gradual formation of GS-SG over time, potentially catalysed by aluminium. Unlike Cu and Fe that are primarily engaged in oxidation phenomena, aluminium plays a less direct role but may still contribute to oxidation (Versari et al., 2023). Although further testing would be required, these factors could collectively explain the remarkable high levels of GS-SG observed in canned samples, particularly between 3 and 6 months (Fig. 1a and b). In the co-spiked groups (Sh+ and Ch+, Fig. 1c and d), GS-SG formed rapidly with supplemented GSH, with the declining trends after 6 months possibly being attributable to the proposed sulfonation/sulfitolysis reactions involving SO_2_ (Arapitsas et al., 2016; Bekker, Kreitman, et al., 2018). Moreover, the relatively stable SO_2_ levels (Table 1) suggested that metal ions released from the packaging may have contributed to the transformation of GS-SG, although this warrants further study.

#### 3.2.3 Polysulfide behaviour over time

**Fig. 2:**
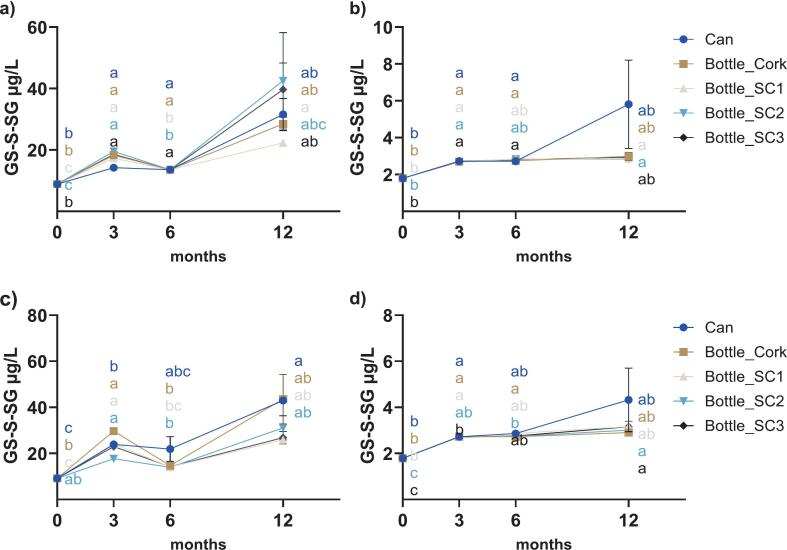
Temporal evolution of GS-S-SG concentrations (μg/L) during ageing in different packaging formats for a) Sh, b) Ch, (c) Sh+, d) Ch+. Error bars represent standard error from duplicate measurements of each sample. Different lowercase letters in each panel (coloured to match the respective package according to the legend) designate significant differences over 12 months within the same package format (from two-way ANOVA on log_10_-transformed data, followed by Tukey's multiple comparison test, α = 0.05). SC, screw cap 1, 2, or 3; refer to Section 2.2 for details.

**Fig. 3:**
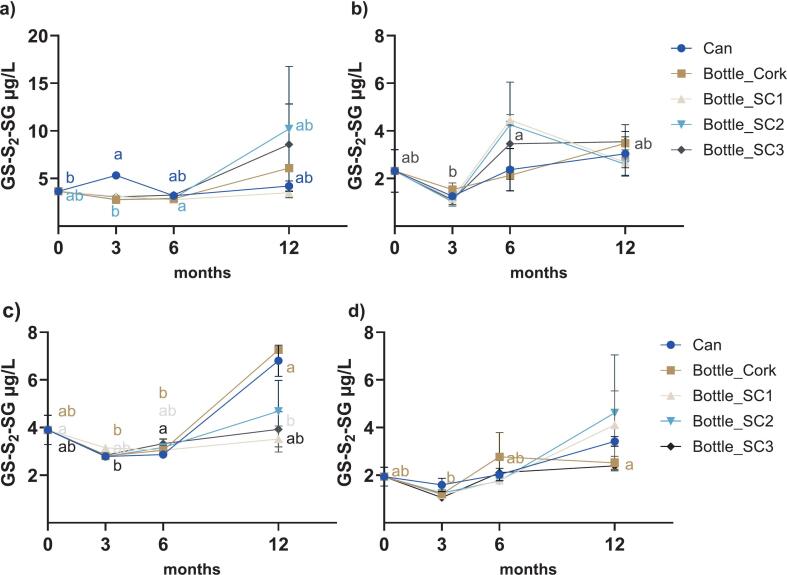
Temporal evolution of GS-S_2_-SG concentrations (μg/L) during ageing in different packaging formats for a) Sh, b) Ch, c) Sh+, d) Ch+. Error bars represent standard error from duplicate measurements of each sample. Different lowercase letters in each panel (coloured to match the respective package according to the legend) designate significant differences over 12 months within the same package format (from two-way ANOVA on log_10_-transformed data, followed by Tukey's multiple comparison test, α = 0.05). SC, screw cap 1, 2, or 3; refer to Section 2.2 for details.

**Fig. 4:**
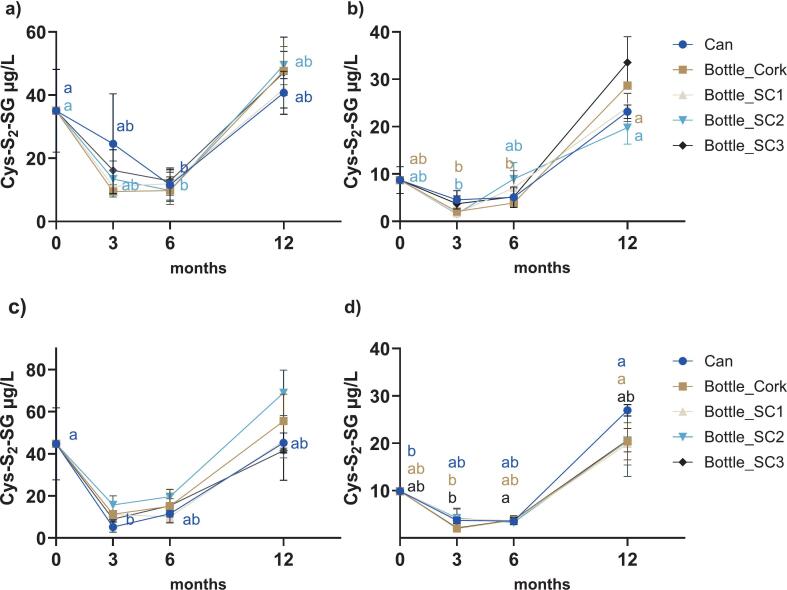
Temporal evolution of Cys-S_2_-SG concentrations (μg/L) during ageing in different packaging formats for a) Sh, b) Ch, c) Sh+, d) Ch+. Error bars represent standard error from duplicate measurements of each sample. Different lowercase letters in each panel (coloured to match the respective package according to the legend) designate significant differences over 12 months within the same package format (from two-way ANOVA on log10-transformed data, followed by Tukey's multiple comparison test, α = 0.05). SC, screw cap 1, 2, or 3; refer to Section 2.2 for details.

GS-S-SG, GS-S_2_-SG, and Cys-S_2_-SG were key polysulfides monitored over the 12-month ageing period (Fig. 2, Fig. 3, Fig. 4). No significant effects of package nor time × package interaction were observed in either variety (*P* ≥ 0.1171) except for GS-S-SG in Sh+ (*P* = 0.0213 for time × package), with time being a significant factor in all cases (*P* ≤ 0.0375, Table A.1, Appendix A). Significant accumulation of GS-S-SG was observed in both control and co-spiked wines under various packages except for Sh+ sealed under SC3 (*P* ≥ 0.0532). Concentrations rose from 8.9 μg/L to ≥22.3 μg/L in Sh (Fig. 2a), 1.8 μg/L to ≥2.8 μg/L in Ch (Fig. 2b), 9.2 μg/L to ≥25.9 μg/L in Sh+ (Fig. 2 c), and 1.8 μg/L to ≥2.9 μg/L in Ch+ (Fig. 2d). Overall, the package appeared to have no effect on GS-S-SG, except at 3 months in Sh (*P* = 0.0002, can vs SC1). Two-way ANOVA between control and co-spiked treatments (Table A.2, Fig. 2a vs 2c and Fig. 2b vs 2d) indicated that GSH/H_2_S supplementation did not significantly impact the levels of GS-S-SG, with the exception of Sh under SC1 (P = 0.0002).

Tetrasulfide GS-S_2_-SG (Fig. 3) was generally lower in concentration than the trisulfide and remained unaffected by packaging type or any interaction of package with time for control and co-spiked groups (Table A.1, Appendix A). In contrast, there was a significant effect of time for both groups (*P* values from 0.0020 to 0.0357). The concentration of GS-S_2_-SG ranged from 3.7 μg/L at the beginning to ≥3.5 μg/L in Sh (Fig. 3a), with significant differences only detected in samples sealed in can and under SC2. In Ch, significant differences were only found under SC3 between 3 and 6 months, where levels varied from an initial 2.3 μg/L to 3.5 μg/L at 12 months (Fig. 3b). In the co-spiked group, GS-S_2_-SG increased from 3.9 μg/L up to 7.3 μg/L in Sh+ (Fig. 3c), albeit without a significant difference detected for can or SC2 closure, and from 1.9 μg/L in sample sealed under cork to 4.1 μg/L in Ch+ with significant differences between 3 and 12 months (Fig. 3d). Overall, the package appeared to have no effect on GS-S_2_-SG, except at 3 months in Ch+ (*P* = 0.0210, SC1 vs SC3). Differences between time points for a given package type were predominantly detected in Shiraz (Fig. 3a and c) rather than Chardonnay (Fig. 3b and d). The co-spiking treatment did not result in a significant difference in GS-S_2_-SG in either variety (*P* ≥ 0.0837) (Table A.2).

The mixed tetrasulfide Cys-S_2_-SG also displayed a significant time effect only (*P* < 0.0001 to *P* = 0.0062), showing no influence of package or its interaction with time for control and co-spiked wines (Table A.1, Appendix A). Sh and Sh+ started with relatively high concentrations (35.0 μg/L and 44.7 μg/L, respectively), with a decrease before ending with higher levels. Significant differences were observed between 0 and 6 months in Sh, with final levels of 40.7 μg/L (can) and 49.5 μg/L (SC2) (Fig. 4a), and between 3 and 12 months in Sh+, ending with levels of 55.6 μg/L (cork) and 69.0 μg/L (SC2) (Fig. 4c). Ch and Ch+ (Fig. 4b and d) had lower initial concentrations (≤9.9 μg/L), but followed a similar trend over time to the red wine counterparts, with a decrease before ending at ≥19.8 μg/L. Significant differences were observed between 0 and 3 months in canned Ch and between 0 and 12 months in canned Ch+, and between 3 and 12 months in Ch+ under cork and SC3. There were no effects of package on Cys-S_2_-SG and its concentration remained unaffected by co-spiking treatment across all packages for each wine variety (*P* ≥ 0.0979) (Table A.2).

#### 3.2.4 Formation and persistence of polysulfides

Compared to disulfide accumulation, the pattern for polysulfides suggested differences in the formation pathways, as discussed by Kreitman et al. (2017), and/or stability profiles as outlined by Hou et al. (2025a). Although oxygen levels were expected to vary due to the different package formats, the lack of significant differences among the package types indicated that oxygen was not a key factor in governing polysulfide formation under the test conditions. Furthermore, while GSH and H_2_S supplementation introduced additional sulfhydryl precursors into the pool, that treatment did not appear to greatly influence any of the quantified polysulfides. This indicated that the polysulfides already present in the wines were the determining factor and that GSH (and H_2_S) contributed to other reactions, such as disulfide formation, rather than being effectively incorporated as sulfane sulfur to build up longer-chain structures. This could imply that polysulfides are not a sink for VSCs such as H_2_S in the long term, even in the presence of metals such as Cu.

Polysulfide concentrations in a set of commercial Australian wines of different vintages were recently reported, with the GS-S-SG levels observed in the present study within the range of that previous work (Hou et al., 2025b). The concentration range of Cys-S_2_-SG also aligned with the published data (Hou et al., 2025b). On the other hand, while GS-S_2_-SG was previously detected in wine (Dekker, Fedrizzi, van Leeuwen, Roman, et al., 2022; Nardin et al., 2020; van Leeuwen et al., 2020), no studies to date have apparently reported its absolute or semi-quantitative concentrations. In the current work, this symmetrical GSH tetrasulfide was detected at up to 10 μg/L, with higher levels in Shiraz than Chardonnay. Research has shown that GS-S-SG and GS-S_2_-SG degraded rapidly in aqueous solution (half-life ≤9 h at room temperature), and Cys-S_2_-SG declined by approximately 60 % after 40 days (Hou et al., 2025a). Nevertheless, all three polysulfides were persistent during the course of the present study, suggesting greater stability in real wine conditions. This aligned with the changes in Cys polysulfide concentrations in model wines stored up to 6 months by Bekker, Kreitman, et al., 2018.

As discussed for the stability of polysulfides in an aqueous system (Hou et al., 2025a), the relative persistence of polysulfides in wine suggested the matrix may slow their degradation, likely due to the low pH, with the potential for interactions with endogenous compounds such as polyphenols, bisulfite, sulfhydryls, and amino compounds. Indeed, polysulfide degradation and formation could occur simultaneously leading to a dynamic chemical equilibrium (Dekker et al., 2023). It is also known that polysulfides of different length have certain distributions, with shorter chains tending to be formed in greater amounts in model wines and synthetic solution (Bekker, Kreitman, et al., 2018; Hou et al., 2025b; Kreitman et al., 2019). Notably, no long-chain polysulfides (*n* ≥ 5) were detected in the present work, although earlier investigation found these species to be relatively stable under aqueous conditions (Hou et al., 2025a). The study of Dekker et al. (2023) highlighted that a pentasulfide was detectable in wine within the first 300 h. In the present case, however, longer chain polysulfides may have degraded below detectable levels during storage, which would accord with the results from a survey of 78 commercial wines, where polysulfides up to tetrasulfide were quantified (Hou et al., 2025b).

### 3.3 Interrelationship between sulfur compounds

Various sulfur-containing compounds coexist in wine and some may play a role in the evolution of di- and polysulfides over time (Bekker, Wilkes, & Smith, 2018). Considering their redox reactivity, H_2_S, SO_2_, and GSH were monitored over the 12-month ageing experiment, with their concentrations summarised in Table 1. H_2_S and GSH can participate in redox reactions (including thiol-disulfide exchange and/or metal-binding reactions) that generate di- and polysulfides, whereas SO_2_ serves as an antioxidant additive and can react with sulfides through sulfitolysis (Bekker, Kreitman, et al., 2018; Kritzinger et al., 2013). In addition, two other VSCs related to reductive characters, namely MeSH and DMS, were also monitored (Table A.3, Appendix A). The DMS formation pathway differs to other fermentative VSCs (Waterhouse et al., 2024d), and lacking a sulfhydryl group, DMS does not partake in polysulfide formation. Nonetheless, it is known to accumulate during storage (Segurel et al., 2005; Siebert et al., 2010) and was included for that reason.

Two-way repeated measures ANOVA (Table A.4, Appendix A) was conducted to investigate the effects of time, package, and their interaction for the analytes listed in Table 1 and Table A.3. Time was the main factor for three VSCs in both varieties (*P* ≤ 0.0198), except for MeSH in the Sh and Sh+, which remained unaffected by time (*P* ≥ 0.0731), package (*P* ≥ 0.2883), or their interaction (*P* ≥ 0.6965). SO_2_ and GSH were also mainly affected by time (*P* ≤ 0.0097) in both varieties. In addition, package and time × package only affected SO_2_ (*P* ≤ 0.0047) in two Shiraz groups, and MeSH in Ch (*P* = 0.0422 and *P* = 0.0003, respectively).

#### 3.3.1 Changes in SO_2_, VSCs and GSH

According to the results in Table 1, SO_2_ declined markedly in Shiraz, from an initial 87.5 μg/L to ∼6 μg/L after 12 months, but was maintained at relatively high levels (≥131 μg/L) throughout ageing in Chardonnay from a starting level of 159 μg/L. Significant differences were observed over the 12 months in certain package formats (Table 1). Package-related differences were only observed at isolated time points: namely in canned Sh at 6 months vs SC1 and SC2 (*P* ≤ 0.0459) and 12 months vs SC3 (*P* = 0.0343), in Sh+ under SC2 at 3 months vs SC3 (*P* = 0.0381), and in canned Ch+ at 12 months vs cork (*P* = 0.0193) (not all data shown). H_2_S levels generally declined over the ageing period, with the most pronounced diminishment detected in Shiraz (from ∼3.8 μg/L to below 1.4 μg/L). In Chardonnay, the change in H_2_S with time was less consistent and greater replicate variation (reflected by higher SE values) was observed (Table 1). On the other hand, differences according to package were observed at certain time points, those being Sh at 6 months (can vs cork, *P* = 0.0216), Ch at 6 months (can vs SC2, *P* = 0.0474) and at 12 months (SC1 vs SC2, *P* = 0.0177), and Ch+ at 6 months (SC1 vs Cork and SC2, *P* ≤ 0.0476) (not all data shown).

GSH concentrations presented different profiles during ageing in control and co-spiked groups for both wine varieties (Table 1). In the control groups, GSH exhibited an overall increasing trend over time for the different package types in Sh (from below 0.1 mg/L to above 0.3 mg/L), and Ch presented a sharp initial increase followed by a decline, resulting in an overall upward trend. In the co-spiked groups, rapid declines were observed (from ∼55 mg/L to below 3.1 mg/L). Significant pairwise differences were observed in Sh, Sh+ and Ch+, while none were observed in Ch (Table 1). Generally, no significant package-related effect was observed between canned and bottled wines, with some exceptions in the co-spiked red wine. At 3 months, Sh+ under cork showed significant differences compared to can, SC1, and SC2 (*P* ≤ 0.0467), and canned Sh+ differed to SC 1 (*P* = 0.0212) (not all data shown).

Regarding the other two VSCs, MeSH and DMS exhibited distinct patterns during ageing (Table A.3, Appendix A). MeSH remained at stable levels (≤2.9 μg/L) in Sh, with no significant differences observed across time points. In Sh+, MeSH was ≤2.9 μg/L, with significant decreases observed in samples under cork and SC2 before returning to initial levels. Beginning at 8.2 μg/L, MeSH in Ch also appeared to decrease before ending no different from the starting amount, although no significant difference was detected for cork, SC1, or SC2. MeSH in Ch+ began at around 2.8 μg/L and tended to experience a slight increase, although there was no significant change for Ch+ sealed under cork. No significant differences (*P* ≥ 0.0931) were observed for the two varieties packaged in can or bottle with the different closures during ageing (data not shown) except that the canned sample initially differed to all SCs (*P* < 0.0001) for Sh and Ch+ .

DMS concentrations accumulated across all the samples, with significant differences observed in Sh+ under cork at 6 vs 12 months (*P* = 0.0286) and most Chardonnay samples except for Ch under cork (*P* ≥ 0.0871) and Ch+ in can (*P* ≥ 0.1302) and under SC3 (*P* ≥ 0.1729). Packaging format had minimal effect on DMS (not all data shown), with an exception at specific time points. At the initial stage, the canned sample initially differed to all SCs (*P* < 0.0001) in all samples except for Ch. Additionally, Ch sealed under SC1 significantly differed from the canned sample (*P* = 0.0030) and from SC2 (*P* = 0.0018) at 6 months, and Ch+ under cork differed from SC2 (*P* = 0.0211) at 3 months.

The changes observed for these sulfur compounds were compared with previous studies. The behaviour of key VSCs was broadly consistent with published data (Bekker, Wilkes, & Smith, 2018; Montgomery et al., 2023; Ugliano et al., 2011; Ugliano et al., 2012). H_2_S showed clear varietal differences, declining in Sh and Sh+ and increasing in Ch and Ch+ (Table 1). Packaging outcomes were also in line with the report by Montgomery et al. (2023), showing substantially higher H_2_S accumulation in can than bottle, while closure type had no significant effect. Other changes aligned with earlier reports, with MeSH remaining stable, DMS accumulating, and SO_2_ decreasing over time (Arapitsas et al., 2016; Bekker et al., 2021; Franco-Luesma & Ferreira, 2016; Gambuti et al., 2020; Zhang et al., 2022; Zhang et al., 2019). GSH is generally reported to decline during wine ageing, which contrasts with the current findings in control wines but aligns with the trends observed in GSH-supplemented wines (Table 1). This variation may be attributed to the relatively low GSH concentrations in control groups (at μg/L range), where observed changes may be within the margin of analytical uncertainty. In contrast, the co-spiked samples where GSH was present in the mg/L range showed significant declines over time as previously reported.

#### 3.3.2 Correlations between volatile and non-volatile sulfur compounds

**Fig. 5:**
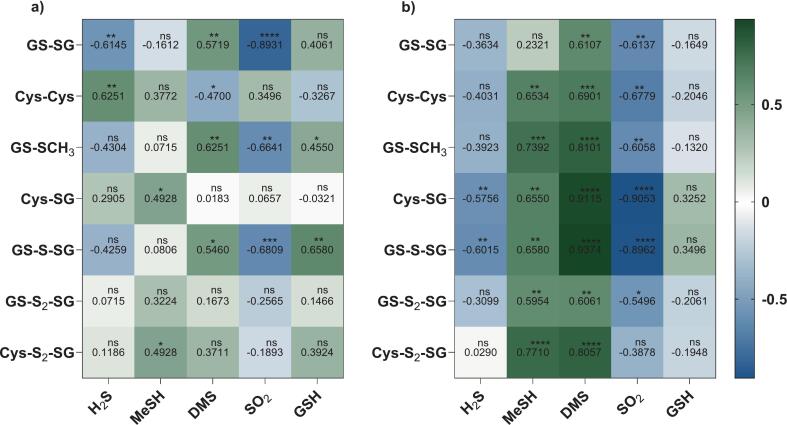
Spearman correlation coefficients and level of significance between redox-related compounds and disulfides/polysulfides in a) Sh and b) Ch considering all time points during 12 months of ageing. Significance levels are represented as follows: *, *P* < 0.05; **, *P* < 0.01; ***, *P* < 0.001; ****, *P* < 0.0001; “ns” denotes comparisons that were not significant.

Correlation analyses were conducted to explore the relationships between polysulfides and related sulfur compounds. Although the wine matrix is inherently complex and correlations do not imply direct causation, the observed associations could provide insights into interactions among these sulfur species. Significant correlation coefficients are summarised in a heatmap (Fig. 5). H_2_S, a known sulfur donor in wine (Müller et al., 2022), was significantly correlated with three disulfides and with GS-S-SG. Specifically, H_2_S was negatively correlated with GS-SG in Sh (Fig. 5a) and with Cys-SG and GS-S-SG in Ch (Fig. 5b). In contrast, Cys-Cys in Sh showed a significant positive correlation with H_2_S (Fig. 5a). These opposing trends may reflect that Cys-Cys formation occurred more readily where H_2_S remained measurable over time, or that Cys polysulfides have a greater propensity to extrude H_2_S. This behaviour may be interpreted as evidence that GSH substitution may provide greater steric protection against nucleophilic attack than Cys substitution (Kreitman et al., 2017; Müller et al., 2022), consistent with previous findings of stable mixed Cys-GSH polysulfides (Hou et al., 2025a).

**Fig. 6:**
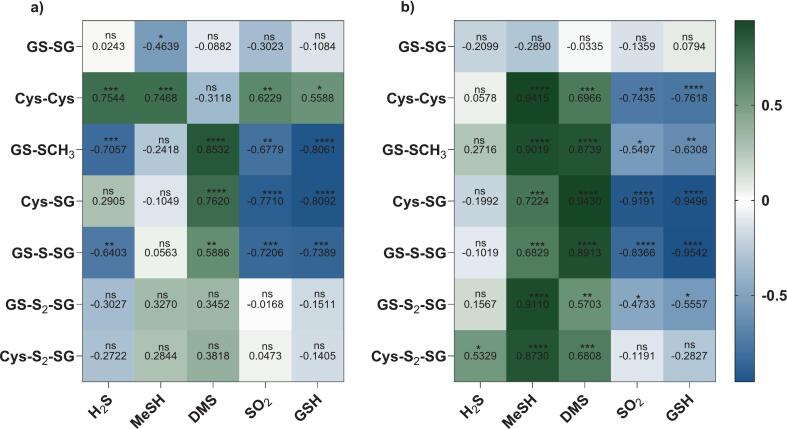
Spearman correlation coefficients and level significance between redox-related compounds and disulfides/polysulfides in a) Sh+ and b) Ch+ considering all time points during 12 months of ageing. Significance levels are represented as follows: *, P < 0.05; **, P < 0.01; ***, P < 0.001; ****, P < 0.0001; “ns” denotes comparisons that were not significant.

Regarding correlations for co-spiked wines, the elevated H_2_S level in these samples introduced different patterns as shown by the significant correlations in Fig. 6, which were mostly found for Sh. In particular, H_2_S remained positively correlated with Cys-Cys like the control (Fig. 5a), while exhibiting negative correlations with GS-SCH_3_ (Fig. 6a). The differences compared to control indicated that spiking with H_2_S (perhaps in conjunction with GSH) perturbed the original sulfur status. Thus, although disulfide formation does not necessarily require the direct involvement of H_2_S, the observed correlations may reflect broader redox dynamics and sulfhydryl-disulfide-polysulfide interconversions within the wine matrix (Hou et al., 2025a; Kreitman et al., 2019). Notably for polysulfide species, the negative correlation between H_2_S and GS-S-SG was observed in both white and red wines, reaching statistical significance in Sh+ and Ch, but not in Sh or Ch+. In contrast, GS-S_2_-SG had no significant correlations with H_2_S across control (Fig. 5) and co-spiked groups (Fig. 6), whereas Cys-S_2_-SG exhibited a significant positive correlation only for Ch+ (Fig. 6b). Together, the results showed that longer-chain polysulfide evolution responded differently to relatively low concentrations of H_2_S, potentially in relation to rates of reaction, the relative concentrations of free sulfhydryls, and matrix components such as polyphenols (which are abundant in red wines).

Significant correlations between MeSH or DMS and the di/polysulfides shown in Fig. 5 were generally positive and particularly strong in Ch (*r* ≥ 0.60). An exception was observed for Cys-Cys, which was negatively correlated with DMS in Sh (*r* = −0.47, *P* = 0.0365). Although not related to polysulfide formation as noted earlier in this section, DMS had stronger associations than MeSH across multiple compounds (especially Cys-SG and GS-S-SG in Ch), likely aligning with its more pronounced increment over ageing (Table A.1, Appendix A). In contrast, MeSH increased to a greater extent in Ch (Table A.1), consistent with the greater number and higher significance of correlations observed in this white wine (Fig. 5b). In Ch, co-spiking treatment produced MeSH and DMS patterns similar to the control group (Fig. 6b vs 5b), except for GS-SG. In Sh+, MeSH showed more and stronger correlations with GS-SG and Cys-Cys (Fig. 6a vs Fig. 5a), while DMS displayed greater positive correlations with GS-CH_3_, Cys-SG, and GS-S-SG but no significant correlation with GS-SG and Cys-Cys compared to Sh. Mechanistically, MeSH is formed “de novo” from precursors such as methionine via chemical or non-enzymatic degradation during ageing, whereas DMS is attributed primarily to the slow hydrolysis of *S*-methyl methionine leading to the gradual increase over time (Ailer et al., 2022; Franco-Luesma & Ferreira, 2016; Goulioti et al., 2025; Jiménez-Lorenzo et al., 2022). The strong correlations suggested that these two VSCs may be favoured by similar underlying factors that influence polysulfide chemistry, such as time and redox environment, rather than direct involvement in polysulfide reactions.

SO_2_ acts both as an antioxidant (reducing agent) and a nucleophile in wine (Waterhouse et al., 2024b), with di- and polysulfides undergoing sulfitolysis reaction with SO_2_ in the latter case (Arapitsas et al., 2016; Bekker, Kreitman, et al., 2018; Kreitman et al., 2019). Consistent with the sulfitolysis mechanism, significant negative correlations were observed between SO_2_ and several sulfide species in Fig. 5, Fig. 6. Among detected disulfides in Sh, significant negative correlations were observed for GS-SG and GS-SCH_3_ (Fig. 5a), whereas in Sh+, there were GS-SCH_3_ and Cys-SG, with GS-SG showing a negative trend that did not reach significance (Fig. 6a). In Ch and Ch+, all disulfides were negatively correlated to SO_2_ except for GS-SG (Fig. 5b and 6b). GS-S-SG was the most consistently correlated polysulfide to exhibit significant associations, with a strong negative correlation with SO_2_ in both varieties under control and co-spike conditions (*r* ≤ −0.68, *P* ≤ 0.001). In contrast, GS-S_2_-SG was negatively correlated with SO_2_ only in Ch and Ch+. Although there were higher initial levels of SO_2_ in the white wine together with less decline during ageing (Table 1), the correlation strength was comparable between the two varieties. This indicated the ability of SO_2_ to cleave the sulfane sulfur bonds in polysulfides, leading to their sulfur chain breakdown (Müller et al., 2022). The weak and non-significant correlations observed for tetrasulfides could potentially be due to their lower concentrations rather than different reactivity towards SO_2_, considering the previous evidence for sulfitolysis of Cys polysulfides in model wine (Bekker, Kreitman, et al., 2018).

When examining the relationships between GSH and di/polysulfides, there were significant positive correlations for GS-SCH_3_ and GS-S-SG (Fig. 5a). Conversely for Ch, GSH was weakly and negatively correlated with GS-SG (Fig. 5b). Given the low initial GSH level in the controls (approximately 100 μg/L, Table 1), further insight was gained from correlation analysis for co-spiked samples (Fig. 6). Under these conditions, correlation patterns shifted markedly, with Cys-Cys in Sh+ displaying a significant positive correlation with GSH (Fig. 6a). In contrast, GSH showed strong negative correlations (*r* ≤ −0.74, *P* ≤ 0.0002) with GS-SCH_3_, Cys-SG, and GS-S-SG (Fig. 6a). In Ch+, GSH exhibited uniformly strong negative correlations (*r* ≤ −0.56, *P* ≤ 0.011) with most of the di- and polysulfide species (Fig. 6b), excluding GS-SG and Cys-S_2_-SG. Interestingly, GS-SG showed a weaker (and non-significant) correlation with GSH than expected (*r* = 0.08, *P* = 0.7394) in Ch+, possibly due to its substantial accumulation up to 6 months and decline thereafter to original levels in co-spiked wines (Table 1). When considering only the first 6 months of ageing in Ch+, a moderate and significantly negative correlation (*r* = −0.58, *P* = 0.023) emerged, aligning with observed negative trends in control Ch (Fig. 5b).

These results demonstrated that higher GSH levels (perhaps in conjunction with the added H_2_S) led to more pronounced and distinct correlation patterns compared to control groups, underscoring the dual roles of GSH in wine. On one hand, GSH can function as the precursor in di- and polysulfide formation as supported by the negative correlations (Fig. 5 and Fig. 6). On the other hand, it may result from di- and polysulfide cleavage according to proposed mechanisms of sulfitolysis or nucleophilic reaction with sulfhydryls (including persulfides) (Bekker, Kreitman, et al., 2018; Kreitman et al., 2019). Thus, despite the well-established redox relationship between GSH and GS-SG (Kritzinger et al., 2013), there can be multiple competing pathways of redistribution of sulfur compounds that collectively disrupt the typical relationship between GSH and GS-SG.

Correlations between polysulfides and disulfides were also explored, revealing matrix-dependent patterns between the two varieties (not all data shown). In Sh, GS-SG showed a significant positive correlation with GS-S-SG (*r* = 0.70, *P* = 0.0006), whereas in Ch, all disulfides except GS-SG correlated strongly with the sets of polysulfides (*r* ≥ 0.48, *P* ≤ 0.0446). For the polysulfides, positive correlations between GSH tetrasulfide and other polysulfides were more pronounced in Ch, while in Sh, significance was observed only with Cys-S_2_-SG. The positive association between GS-S-SG and GS-S_2_-SG was consistent with the previous study (Hou et al., 2025a), yet correlations involving GS-SG differed from those findings in aqueous system, likely reflecting the complex influences of a wine matrix.

### 3.4 Changes under accelerated ageing

Accelerated shelf-life testing, in which products are stored at higher temperatures or other extremes, is widely used in the testing of food packaging (Montgomery et al., 2023). In the present case, ampoule-based accelerated ageing was used to probe the potential reactivity of sulfur compounds that might provide insights for longer term natural ageing (Casassa et al., 2023; Goulioti et al., 2025). Fig. A.4 for Sh and Fig. A.5 for Ch (Appendix A) show detected sulfur compounds with significant differences among ageing conditions relative to their initial levels, including control wines under SC1 (lowest OTR for bottled wine) and canned wine under real-time ageing for a comparison with ampouled and canned wine upon accelerated ageing (labelled with lower case letters).

Among the nine compounds that were significantly different by one-way ANOVA in Sh, accelerated ageing generally promoted declines. Exceptions were DMS (141 % increase) and GSH (22 % increase) in canned wines, and GS-S_2_-SG (2 % increase) in ampouled wines (Fig. A.4, Appendix A). The effects of ageing conditions were package-dependent. Significant differences between real-time and accelerated ageing were observed only in canned wines including H_2_S (Fig. A.4a, *P* = 0.020), GS-SCH_3_ (Fig. A.4b, *P* = 0.0084) and GS-S_2_-SG (Fig. A.4c, *P* = 0.016). In contrast, a significant loss of SO_2_ (*P* = 0.0009) was only detected in glass format group under accelerated ageing (Fig. A.4d). Significant differences were found for both packaging types for GSH (Fig. A.4e), the disulfides Cys-Cys and Cys-SG (Fig. A.4f and A.4g) and trisulfide GS-S-SG (Fig. A.4h) when comparing between the two ageing conditions (P = 0.0006 to 0.0403, not all data shown). Under accelerated ageing in Sh, package-related differences (marked with lower case letters) were detected for SO_2_ (Fig. A.4d, *P* = 0.0002), GSH (Fig. A.4e, *P* = 0.0143), and DMS (Fig. A.4i, P = 0.020), all of which were better retained in can. In contrast, GS-S_2_-SG (Fig. A.4c, *P* = 0.030) declined markedly in canned wine relative to ampoule. Notably, DMS showed no effect within can or glass but some variance can be observed (Fig. A.4i).

In Ch, eight analytes changed significantly, with accelerated ageing leading to notable accumulation of H_2_S (up to 369 %) and MeSH (up to 281 %) in canned wine but declines (8 % to near complete loss) in other sulfur compounds (Fig. A.5, Appendix A). Among the two types of ageing, significant differences in H_2_S (Fig. A.5a, P = 0.0002) and MeSH (Fig. A.5b, *P* = 0.0128) were observed only for canned wine. In contrast, significant changes were detected in glass format for SO_2_ (Fig. A.5c, *P* < 0.0001), GSH (Fig. A.5d, *P* = 0.0054), GS-SG (Fig. A.5e, *P* = 0.0101), and Cys-SG (Fig. A.5f, *P* = 0.0476). Both storage formats exhibited significant effects for Cys-Cys and GS-SCH_3_ (Fig. A.5g and A.5h, *P* ≤ 0.014). Beyond differences between ageing condition, a package-specific effect was observed under accelerated ageing (lower case letters, Fig. A.5a–e, *P* ≤ 0.0161). In particular, H_2_S, MeSH, SO_2_, and GSH were significantly higher or better retained (smaller decrease) in can, whereas GS-SG was substantially increased in ampoule.

Co-spiked treatments involving H_2_S/GSH were also examined after accelerated ageing. Significant results are presented in Fig. A.6 (Sh+) and Fig. A.7 (Ch+), with *P*-values for comparisons to the respective controls given in Table A.5 (Appendix A). Co-spiked wines showed overall similar trends to the controls (Fig. A.6 vs Fig. A.4, Fig. A.7 vs Fig. A.5), but with a greater number of significant within- and between-format differences being detected. Notably, the elevated initial GSH level in co-spiked wines led to slightly different results. GSH decreased by 66 %–100 % in Sh+, with its derived disulfide (GS-SG) increasing in ampoule (40 %) but declining in can (up −20 %) (Fig. A.6). Ch+ presented a comparable pattern, with GSH consistently decreasing and GS-SG increasing in ampoule (14 %) but decreasing in can (−39 %). Notably, the storage format effect (marked with lower case letters) in Ch+ was less pronounced and was mainly observed for lower-molecular-weight sulfur compounds (Fig. A.7).

There were limited effects of the co-spiking treatment, although its influence varied among the storage format (can vs ampoule) (Table A.5, Appendix A). Significant differences in canned Shiraz were observed for Cys-Cys (*P* = 0.0375), Cys-SG (*P* = 0.0376), and GS-S_2_-SG (*P* = 0.0029), while in glass, GSH and GS-SG were affected by the co-spiking of GSH/H_2_S (*P* ≤ 0.0286). GS-S-SG was significantly influenced by co-spiking in both packages (*P* = 0.0371 and 0.0298, respectively). In Chardonnay, the effect of co-spiking was restricted to H_2_S in can (*P* = 0.0001), MeSH in glass (*P* = 0.0093), and GSH in both formats (P = 0.0002 and 0.0002 for can and glass, respectively).

Accelerated ageing at ∼38 °C introduced moderate thermal conditions compared to real-time (∼18 °C) storage, with the elevated temperature expected to increase the rate of chemical reactions (Waterhouse et al., 2024e). The results suggest that accelerated ageing amplifies change and provides the likely direction of sulfur compound transformation under real-time ageing. The significant declines of disulfides (Fig. A.4b, A.4f, and A.4 g, and Fig. A.5f–h, Appendix A) and tetrasulfide (Fig. A.4c) under accelerated ageing suggested their relative lack of thermal stability, consistent with a previous report (Hou et al., 2025a). From the package perspective, canned wine appeared to retain VSCs (Fig. A.4i, Fig. A.5a and A.5b) and SO_2_ (Fig. A.4d and A.5c) in Sh and Ch during accelerated ageing, echoing the earlier findings regarding VSC formation in canned wines (Montgomery et al., 2023; Versari et al., 2023). Most disulfides (Fig. A.4b, A.4f, and A.4g, and Fig. A.5f–h) and GSH trisulfide (Fig. A.4h) presented no significant difference between packages under accelerated ageing. A potential preserving effect of can was also observed for antioxidant GSH (Fig. A.4e and A.5d), although this was not evident in the co-spiked group (Fig. A.6e and A.7d) and potentially warrants future investigation.

### 3.5 Impact of redox-related treatments under accelerated conditions

A series of treatments designed to reflect practical winemaking considerations was applied under accelerated ageing conditions to assess their influence on the fate of the measured sulfur compounds. Treatments included the addition of metal ions (Cu or Fe), antioxidants (SO_2_ with/without AA), and sealing under nitrogen (N_2_). Most treatments had no significant effect on measured sulfur species in either wine variety, with the significant results being summarised in Table 2. The N_2_-flushed Sh group significantly differed to ampoules sealed under ambient air (untreated) for H_2_S (smaller decrease, *P* = 0.0486), MeSH (slight increase, *P* = 0.0042), SO_2_ (smaller decrease, P < 0.0001), GSH (slight increase, P = 0.0002), and GS-SCH_3_ (substantial increase, *P* = 0.0004). In Ch, the use of N_2_ significantly affected Cys-Cys (smaller decrease, P = 0.0002) and GS-S_2_-SG (moderate increase, *P* = 0.0147). Addition of SO_2_, either alone or in combination with AA, only affected SO_2_ levels in Sh, resulting in greater depletion than untreated groups (*P* ≤ 0.0027), while no significant differences were observed for antioxidant addition in Ch. Metal-related effects were only apparent in Ch, where Cu addition (4 mg/L) led to substantial declines in H_2_S (*P* = 0.0369), SO_2_ (*P* = 0.0187), and GS-S-SG (*P* = 0.0061).

**Table 2:** One-way ANOVA results with treatment as fixed factor showing the effect of N_2_, SO_2_, SO_2_ + AA, or Cu addition on the change (absolute and relative) compared to initial levels for sulfur compounds in Shiraz and Chardonnay wines under accelerated ageing at ∼38 °C for 5 weeks.

	Untreated (μg/L)	% Change	Treated (μg/L)	% Change	P-value
*N*_*2*_	Sh				
H_2_S	−1.29	−51 %	−0.414	−2 %	0.0486
MeSH	−1.10	−39 %	0.0868	17 %	0.0042
SO_2_	−54.3	−93 %	−19.0	−33 %	<0.0001
GSH	−123	−100 %	33.4	28 %	0.0002
GS-SCH_3_	−8.79	−100 %	77.2	880 %	0.0004
*N*_*2*_	Ch				
Cys-Cys	−1.86	−100 %	−0.389	−21 %	0.0002
GS-S_2_-SG	−0.380	−36 %	0.532	59 %	0.0147
*SO*_*2*_	Sh				
SO_2_	−54.3	−93 %	−72.2	−100 %	0.0018
*SO*_*2*_ *+ AA*	Sh				
SO_2_	−54.3	−93 %	−71.2	−98 %	0.0027
*Cu*	Ch				
H_2_S	0.566	23 %	−1.19	−47 %	0.0369
SO_2_	−71.7	−51 %	−129	−91 %	0.0187
GS-S-SG	1.38	1.3 %	3.83	−29 %	0.0061

Values are expressed as absolute differences and as changes relative to initial levels. Only analytes with significant differences are included.

Wines co-spiked with GSH and H_2_S were also affected by N_2_-flushing, addition of antioxidants (SO_2_ and its co-addition with AA), or Cu, but resulted in slightly different profiles across various sulfur compounds (Table A.6, Appendix A). N_2_-flushed treatment of Sh+ showed an opposite trend to the untreated control for H_2_S (increase vs decrease, *P* = 0.0109), SO_2_ (smaller decrease, P < 0.0001), GS-SCH_3_ (substantial increase, P = 0.0002), and Cys-SG (smaller decrease, *P* = 0.0035). In Ch+, N_2_ also remarkably increased H_2_S and MeSH (*P* ≤ 0.0253), led to smaller decrease in SO_2_ (P = 0.0042), accumulated Cys derived disulfides (*P* ≤ 0.0004), and preserved GS-SCH_3_ (P < 0.0001). Addition of SO_2_ (only in Sh+, P = 0.0001) and its co-addition with AA (P ≤ 0.016) significantly affected SO_2_, consistent with the non-spiked untreated groups. Cu addition resulted in significant differences for disulfides in Sh+ in contrast to Sh, where it substantially promoted the accumulation of GS-SG and Cys-SG (>211 % increase, *P* ≤ 0.0127). In contrast, Cu treatment of Ch+ led to a significant decrease in MeSH (−20 %, *P* ≤ 0.0423) and especially SO_2_ (−98 %, P < 0.0001), but increased the concentration of GS-S-SG (354 %, *P* = 0.0026).

From the perspective of di- and polysulfides, the N_2_-flushed treatment resulted in preservation and/or promoted accumulation (Table 2, Table A.6). Antioxidant treatments (SO_2_, AA, or their combination) had no effect on measured sulfur species except for SO_2_. The post-bottling Cu addition had the potential to mask the VSCs by forming Cu-sulfide species, which are known to be brine-releasable (Kreitman et al., 2017). It also contributed to the loss of SO_2_, particularly in Ch (Table 2) and Ch+ (Table A.6, Appendix A), possibly by direct interaction (Bekker et al., 2016) or catalysing oxygen-mediated reactions that generate highly reactive intermediates (e.g., quinones, hydrogen peroxide), thereby accelerating SO_2_ depletion (Kontoudakis & Clark, 2020). SO_2_-mediated sulfitolysis of polysulfides could introduce H_2_S (Bekker, Kreitman, et al., 2018), and reducing agents such as GSH can also cleave S—S bonds of di- and polysulfides to thiols (Hou et al., 2025a; Nagy, 2013), but these effects were not evident under the chosen conditions.

## 4 Conclusion

The study demonstrated the temporal evolution of key sulfur compounds in two wine varieties under different packaging and storage conditions, with specific focus on di- and polysulfides derived from GSH. GS-SG concentrations increased over time with markedly higher accumulation in aluminium can. Other disulfides did not differ as a function of package type but GS-SCH_3_ increased up to two-fold. There was a significant time effect for the studied polysulfides in some package types, but spiking with GSH and H_2_S had no effect for either wine variety, implying that additional formation of sulfane sulfur compounds did not occur. Differences associated with oxygen availability (based on packaging type) and wine susceptibility to oxidation (white vs red wine) could account for the results, with SO_2_ likely playing a role as antioxidant and source of bisulfite for sulfitolysis reactions.

VSCs were primarily governed by time and wine variety, while no significant effect of package was evident. A key insight was the negative correlation between H_2_S and certain GSH-containing di- and trisulfides, with results likely reflective of concentrations of free sulfhydryls, reaction rates, and components such as polyphenols that form electrophilic oxidation products.

Accelerated ageing experiments pointed to an amplification of changes and may provide an indication of sulfur-compound transformations occurring over time with cellaring. This could be useful when developing a shelf-life assay in relation to SLOs. On the other hand, addition of metal ions or antioxidant, or sealing under nitrogen, generally had little effect on the measured compounds in either wine. Overall, inert conditions could lead to accumulation of volatile sulfhydryls whereas Cu can mask them, but Cu also depletes SO_2_ and can catalyse oxidation reactions.

These findings deliver practical insights for the wine industry. First, the time-dependent evolution of VSCs and di- and polysulfides during ageing provides a potential chemical basis for selecting package aligned with ageing profiles. Second, the seemingly limited efficacy of bottling interventions indicates that sulfur management requires decisive measures during winemaking that limit the concentration of latent forms of VSCs. Future research could focus on VSC management without triggering the formation of polysulfides, with the goal of maintaining aroma integrity over time. Expanding the analysis to include additional wine varieties and alternative packages could further elucidate the occurrence of polysulfides and their relationship with VSCs and wine sensory profiles. This could ultimately inform tailored winemaking and packaging strategies to maximise wine quality and commercial benefit across wine varieties.

## CRediT authorship contribution statement

**Yu Hou:** Writing – review & editing, Writing – original draft, Visualization, Investigation, Formal analysis, Conceptualization. **Marlize Z. Bekker:** Writing – review & editing, Supervision, Funding acquisition, Conceptualization. **Tracey E. Siebert:** Writing – review & editing, Supervision. **Gal Y. Kreitman:** Writing – review & editing, Supervision. **David W. Jeffery:** Writing – review & editing, Supervision, Resources, Project administration, Funding acquisition, Conceptualization.

## Funding sources

This work was partly funded by the University of Adelaide and the Australian Wine Research Institute. Additional support was provided by Wine Australia, with levies from Australia's grapegrowers and winemakers and matching funds from the Australian Government. Y.H. was the recipient of a joint scholarship from the University of Adelaide and Wine Australia (UA Ph2102).

## Declaration of competing interest

The authors declare the following financial interests/personal relationships which may be considered as potential competing interests: Yu Hou reports financial support was provided by Wine Australia. Given their role as a co-guest editor, David W. Jeffery had no involvement in the peer review of this article and had no access to information regarding its peer review. Full responsibility for the editorial process for this article was delegated to another journal editor. The remaining authors declare that they have no known competing financial interests or personal relationships that could have appeared to influence the work reported in this paper.

## Data Availability

All data is reported in the article and provided in the supporting information file

## Appendix A Supplementary data

Supplementary data description Fig. A.1. Experimental design showing treatments, packaging, and ageing regimen; Fig. A.2. Temporal evolution of Cys-Cys, GS-SCH_3_, Cys-SG concentrations for wine in different packaging formats under real-time cellaring; Fig. A.3. Temporal evolution of Cys-Cys, GS-SCH_3_, and Cys-SG concentrations for co-spiked wine in different packaging formats under real-time cellaring. Fig. A.4. Significant changes in the percentage of sulfur compounds in Shiraz under real-time cellaring and accelerated ageing; Fig. A.5. Significant changes in the percentage of sulfur compounds in Chardonnay under real-time cellaring and accelerated ageing; Fig. A.6. Significant changes in the percentage of sulfur compounds in co-spiked Shiraz under real-time cellaring and accelerated ageing; Fig. A.7. Significant changes in the percentage of sulfur compounds in co-spiked Chardonnay under real-time cellaring and accelerated ageing; Table A.1. Two-way ANOVA showing the simple effect of ageing time, package, and their interaction for di- and polysulfides in control and co-spiked Shiraz and Chardonnay wines under real-time cellaring; Table A.2. Two-way ANOVA showing the simple effect of ageing time, package, and their interaction for di- and polysulfides in wines in different packages under real-time cellaring; Table A.3. Concentrations of brine-releasable MeSH and DMS in control and co-spiked wines during real-time cellaring; Table A.4. Two-way ANOVA showing the simple effect ageing time, package, and their interaction for H_2_S, MeSH, DMS, SO_2_, and GSH in control and co-spiked wines under real-time cellaring; Table A.5. One-way ANOVA showing significant P-values for analytes with a significant difference between control vs co-spiked treatments in wines under accelerated ageing; Table A.6. One-way ANOVA showing the effect of N_2_, SO_2_, SO_2_ + AA, or Cu addition on change in sulfur compounds in co-spiked wines under accelerated ageing. Image 1
