# Supplementary material for: Understanding polysulfide evolution in wine: insights from accelerated ageing and real-time cellaring in different packaging
Source: Food Chem X. 2025 Dec 26;33:103447. doi: 10.1016/j.fochx.2025.103447 (PMC12825059; doi:10.1016/j.fochx.2025.103447)
Supplement: Supplementary file 1 — Supplementary data description Fig. A.1. Experimental design showing treatments, packaging, and ageing regimen; Fig. A.2. Temporal evolution of Cys-Cys, GS-SCH3, Cys-SG concentrations for wine in different packaging formats under real-time cellaring; Fig. A.3. Temporal evolution of Cys-Cys, GS-SCH3, and Cys-SG concentrations for co-spiked wine in different packaging formats under real-time cellaring. Fig. A.4. Significant changes in the percentage of sulfur compounds in Shiraz under real-time cellaring and accelerated ageing; Fig. A.5. Significant changes in the percentage of sulfur compounds in Chardonnay under real-time cellaring and accelerated ageing; Fig. A.6. Significant changes in the percentage of sulfur compounds in co-spiked Shiraz under real-time cellaring and accelerated ageing; Fig. A.7. Significant changes in the percentage of sulfur compounds in co-spiked Chardonnay under real-time cellaring and accelerated ageing; Table A.1. Two-way ANOVA showing the simple effect of ageing time, package, and their interaction for di- and polysulfides in control and co-spiked Shiraz and Chardonnay wines under real-time cellaring; Table A.2. Two-way ANOVA showing the simple effect of ageing time, package, and their interaction for di- and polysulfides in wines in different packages under real-time cellaring; Table A.3. Concentrations of brine-releasable MeSH and DMS in control and co-spiked wines during real-time cellaring; Table A.4. Two-way ANOVA showing the simple effect ageing time, package, and their interaction for H2S, MeSH, DMS, SO2, and GSH in control and co-spiked wines under real-time cellaring; Table A.5. One-way ANOVA showing significant P-values for analytes with a significant difference between control vs co-spiked treatments in wines under accelerated ageing; Table A.6. One-way ANOVA showing the effect of N2, SO2, SO2 + AA, or Cu addition on change in sulfur compounds in co-spiked wines under accelerated ageing. [file mmc1.pdf]

Appendix A: Supplementary data

Understanding polysulfide evolution in wine: Insights from accelerated ageing and real-time cellaring in different packaging

Yu Hou[a], Marlize Z. Bekker[b], Tracey E. Siebert[c], Gal Y. Kreitman[d], David W. Jeffery[a, *]

[a] School of Agriculture, Food and Wine, and Waite Research Institute, The University of Adelaide, PMB 1, Glen Osmond, SA 5064, Australia

[b] School of Agriculture and Food Sustainability, The University of Queensland, St Lucia, QLD 4072, Australia

[c] The Australian Wine Research Institute, PO Box 46, Glenside, SA 5064, Australia

[d] Gallo, Modesto, CA 95354, USA

* Corresponding author.

E-mail address: david.jeffery@adelaide.edu.au (D.W. Jeffery).

Table of Contents

	Page
**Fig. A.1.** Experimental design showing wine varieties, treatments, packaging conditions, and ageing strategies.	S2
**Fig. A.2.** Temporal evolution of three disulfide concentrations (µg/L) during ageing in different packaging formats for a) Cys-Cys in Sh, b) Cys-Cys in Ch, c) GS-SCH_3 in Sh, d) GS-SCH_3 in Ch, e) Cys-SG in Sh, and f) Cys-SG in Ch under real-time cellaring.	S3
**Fig. A.3.** Temporal evolution of disulfide concentrations (µg/L) during ageing in different packaging formats for a) Cys-Cys in Sh +, b) Cys-Cys in Ch +, c) GS-SCH_3 in Sh +, d) GS-SCH_3 in Ch +, e) Cys-SG in Sh +, and f) Cys-SG in Ch + under real-time cellaring.	S4
**Fig. A.4.** Significant changes in the percentage of sulfur compounds in Shiraz under real-time cellaring and accelerated ageing.	S5
**Fig. A.5.** Significant changes in the percentage of sulfur compounds in Chardonnay under real-time cellaring and accelerated ageing.	S6
**Fig. A.6.** Significant changes in the percentage of sulfur compounds in co-spiked Shiraz (H_2S/GSH) under real-time cellaring and accelerated ageing.	S7
**Fig. A.7.** Significant changes in the percentage of sulfur compounds in co-spiked Chardonnay (H_2S/GSH) under real-time cellaring and accelerated ageing.	S8
**Table A.1.** Two-way ANOVA showing the simple effect of ageing time, package, and their interaction, showing P-values of di- and polysulfides in control and co-spiked (GSH/H_2S) Shiraz and Chardonnay wines under real-time cellaring.	S9
**Table A.2.** Two-way ANOVA showing the simple effect of ageing time, package, and their interaction, showing P-values of di- and polysulfides in Shiraz and Chardonnay wines in different packages under real-time cellaring.	S10
**Table A.3.** Concentrations of brine-releasable methanethiol (MeSH) and dimethyl sulfide (DMS) in control and co-spiked (GSH/H_2S) Shiraz and Chardonnay wines monitored during real-time cellaring.	S11
**Table A.4.** Two-way ANOVA showing the simple effect ageing time, package, and their interaction, showing P-values of three VSCs (H_2S, MeSH, DMS), SO_2, and GSH in control and co-spiked (GSH/H_2S) Shiraz and Chardonnay under real-time cellaring.	S12
**Table A.5.** One-way ANOVA showing significant P-values for analytes with at least one packaging format showing a significant difference between control vs co-spiked (GSH/H_2S) treatments in Shiraz and Chardonnay under accelerated ageing.	S12
**Table A.6.** One-way ANOVA results showing the effect of N_2, SO_2, SO_2 + AA, or Cu addition on change compared to initial levels for sulfur compounds in co-spiked (H_2S/GSH) Shiraz and Chardonnay under accelerated ageing.	S13

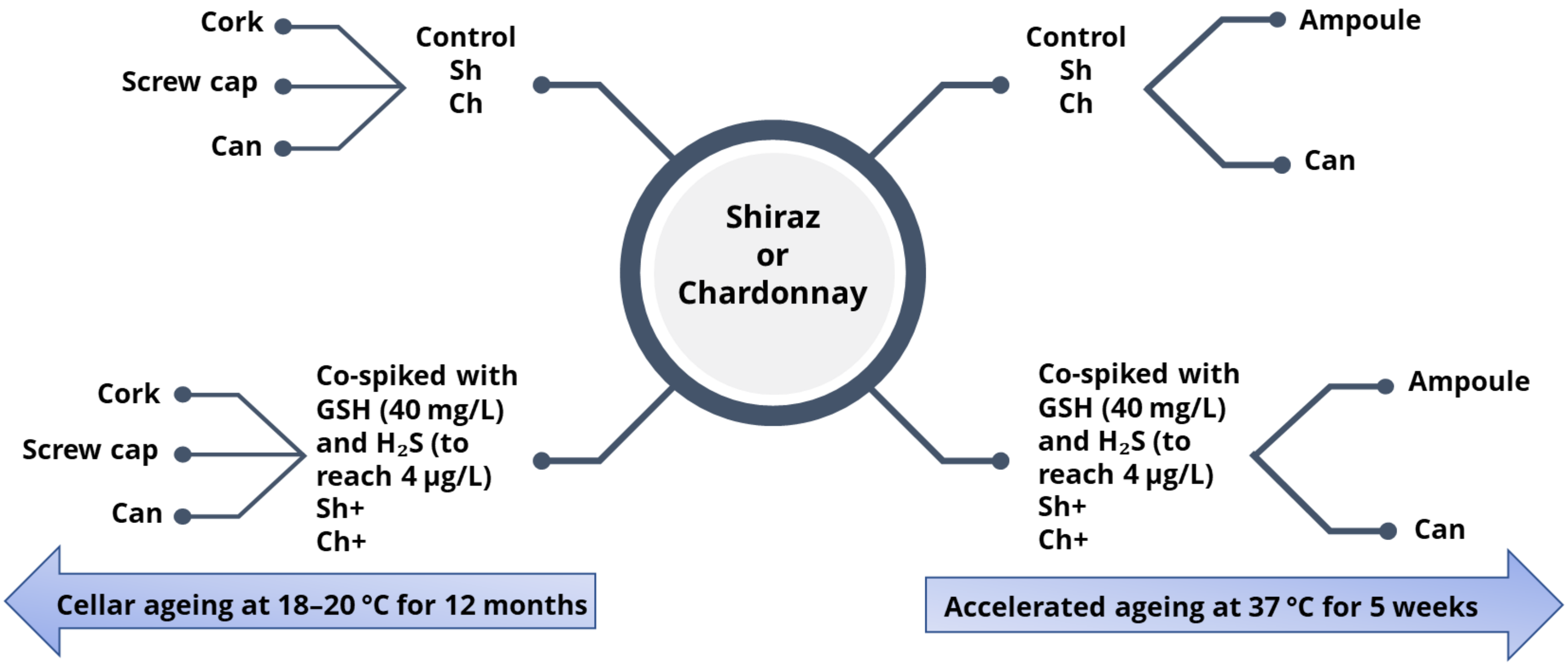

Screw cap	OTR (mL/day/bottle)
SC1	0.002
SC2	0.002–0.005
SC3	0.005

Experimental treatments included filling under N_2, and filling under ambient air (control), and air with the addition of either SO_2, AA, a combination of SO_2 and AA, Fe, Cu.

Fig. A.1. Experimental design showing wine varieties, treatments, packaging conditions, and ageing strategies. Shiraz and Chardonnay wines (control and co-spiked with GSH and H_2S) were stored under cellar ageing (18–20 °C, analysed at t = 0, 3, 6, and 12 months) or accelerated ageing (37 °C for 5 weeks) across different packaging formats (can and bottles with various closures). Each treatment was conducted in duplicate.

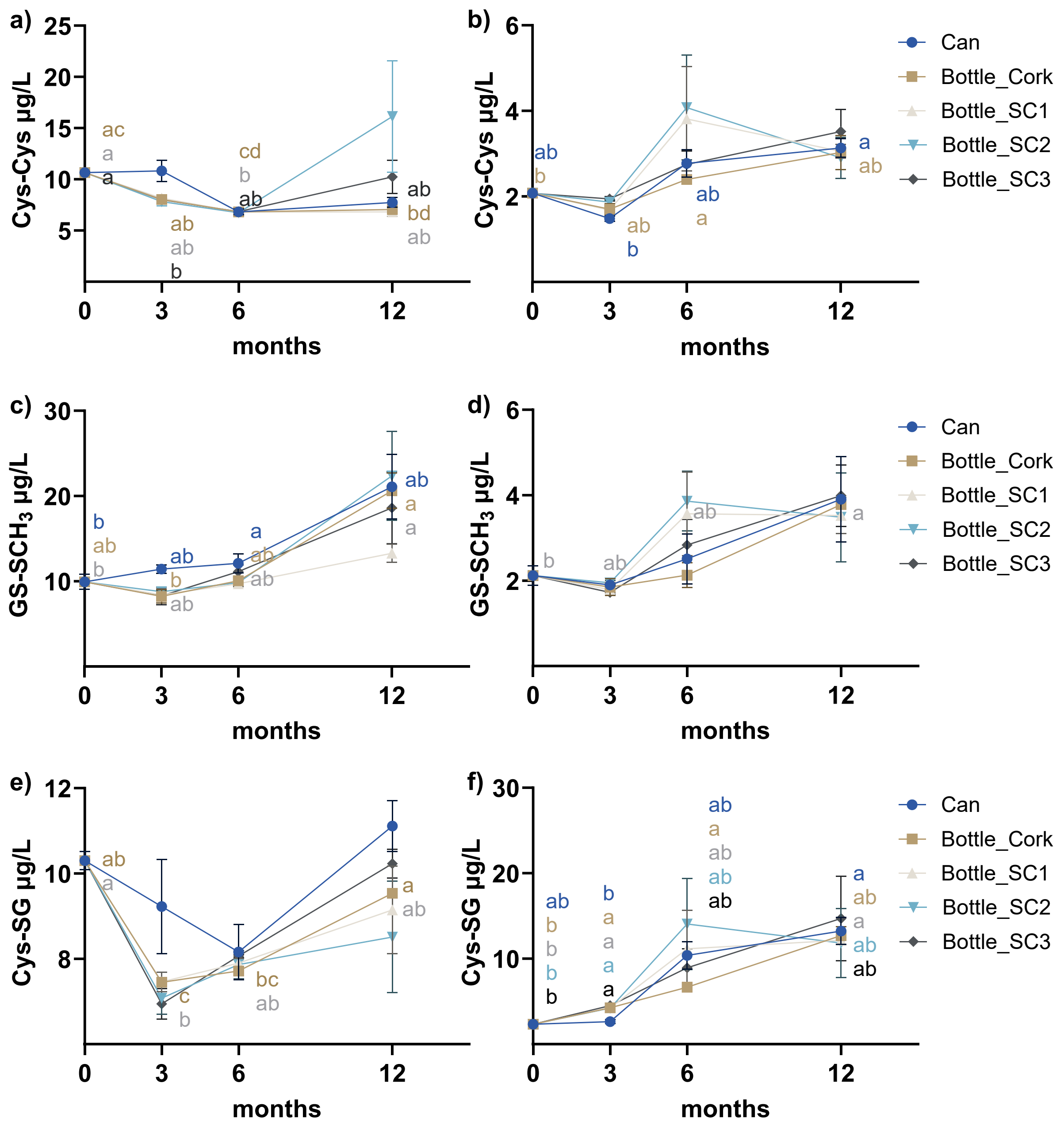

Fig. A.2. Temporal evolution of three disulfide concentrations (µg/L) during ageing in different packaging formats for a) Cys-Cys in Sh, b) Cys-Cys in Ch, c) GS-SCH_3 in Sh, d) GS-SCH_3 in Ch, e) Cys-SG in Sh, and f) Cys-SG in Ch under real-time cellaring. Error bars represent standard error from duplicate measurements of each sample. Different lowercase letters in each panel (coloured to match the respective package according to the legend) designate significant difference over 12 months within the same package format (from two-way ANOVA on log_{10}-trasnformed data, followed by Tukey's multiple comparison test, $\alpha = 0.05$). SC, screw cap 1, 2 or 3; refer to Fig. A.1.

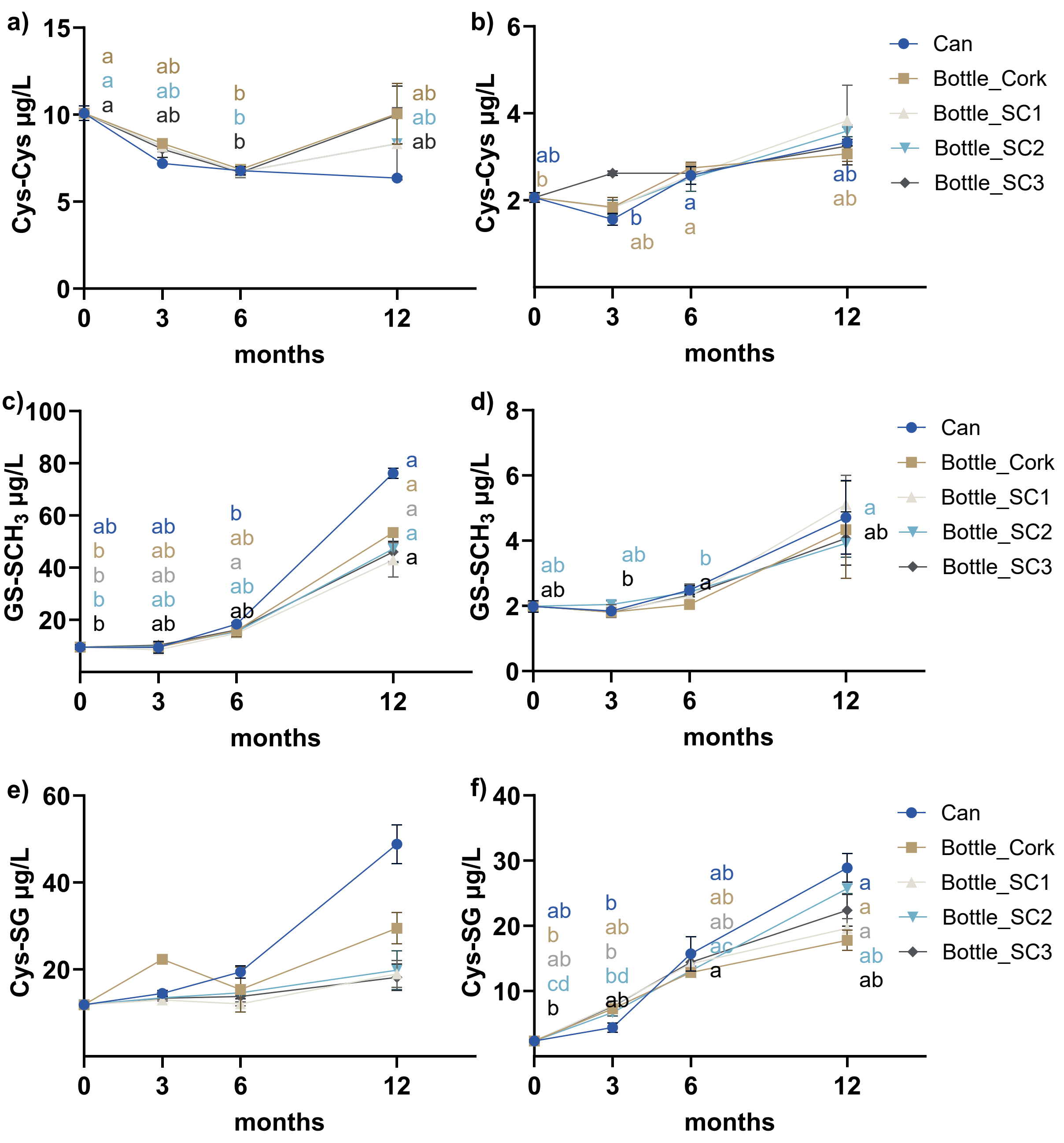

Fig. A.3. Temporal evolution of disulfide concentrations (µg/L) during ageing in different packaging formats for a) Cys-Cys in Sh+, b) Cys-Cys in Ch+, c) $GS\text{-}SCH_3$ in Sh+, d) $GS\text{-}SCH_3$ in Ch+, e) Cys-SG in Sh+, and f) Cys-SG in Ch+ under real-time cellaring. Error bars represent standard error from duplicate measurements of each sample. Different lowercase letters in panel (coloured to match the respective package according to the legend) designate significant difference over 12 months within the same package format (from two-way ANOVA on log10-trasnformed data, followed by Tukey's multiple comparison test, $\alpha = 0.05$). SC, screw cap 1, 2, or 3; refer to Fig. A.1.

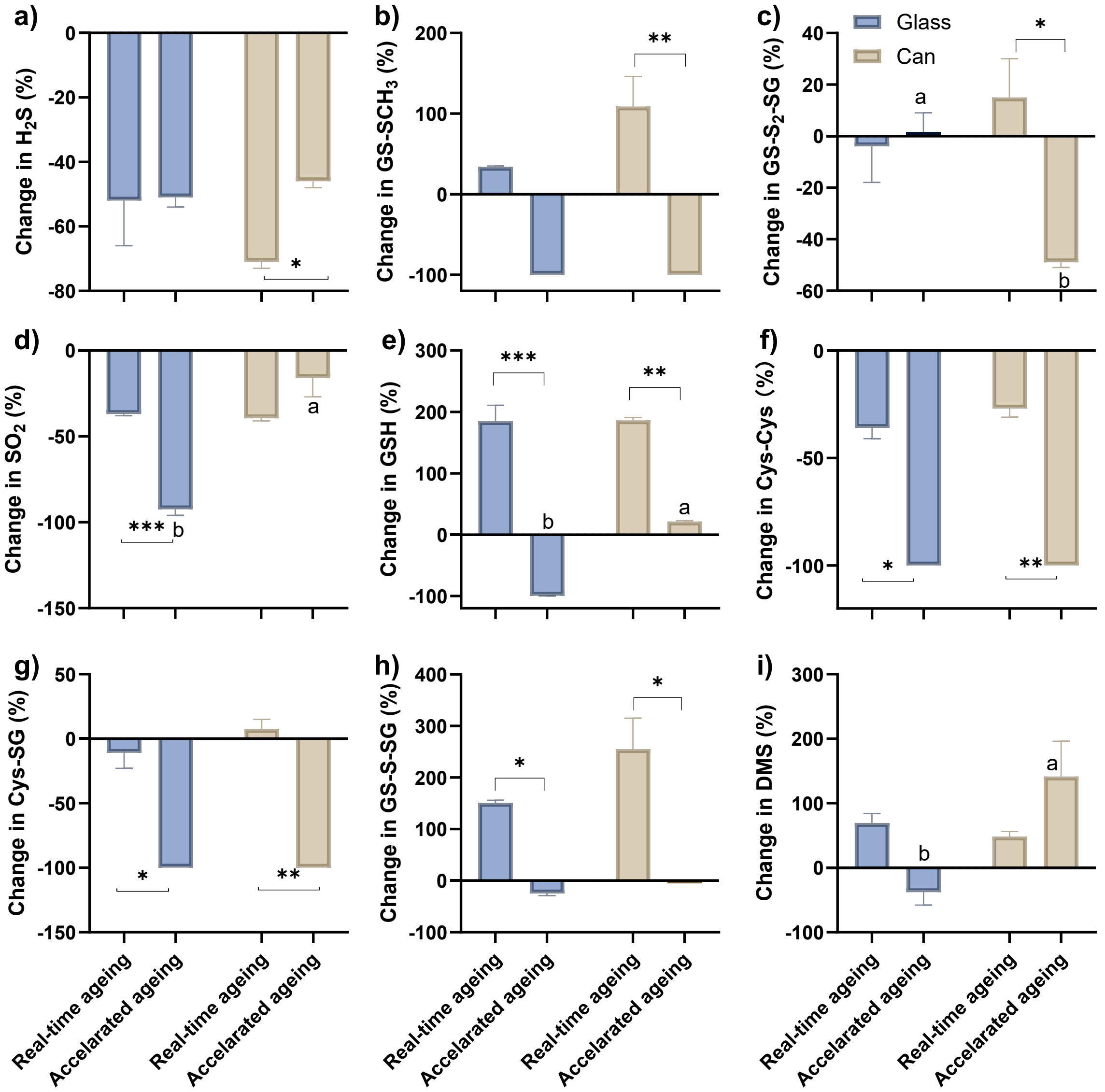

Fig. A.4. Significant percent change of sulfur compounds in Shiraz under real-time and accelerated ageing conditions, expressed relative to initial levels showing a) H_2S, b) GS-SCH_3, c) GS-S_2-SG, d) SO_2, e) GSH, f) Cys-Cys, g) Cys-SG, h) GS-S-SG, and i) DMS. Error bars represent standard error of duplicate measurements. Statistical analyses were performed on raw values (Δ = Tend − T0) whereas the figure displays the corresponding percent changes relative to initial levels. Significant differences were determined by one-way ANOVA followed by Tukey's multiple comparison test (α = 0.05). Asterisks indicate significant differences between ageing conditions within the same package, and different letters above bars indicate significant differences between packaging formats under the accelerated ageing. Significance levels are represented as follows: *, $P < 0.05$; **, $P < 0.01$; ***, $P < 0.001$; ****, $P < 0.0001$.

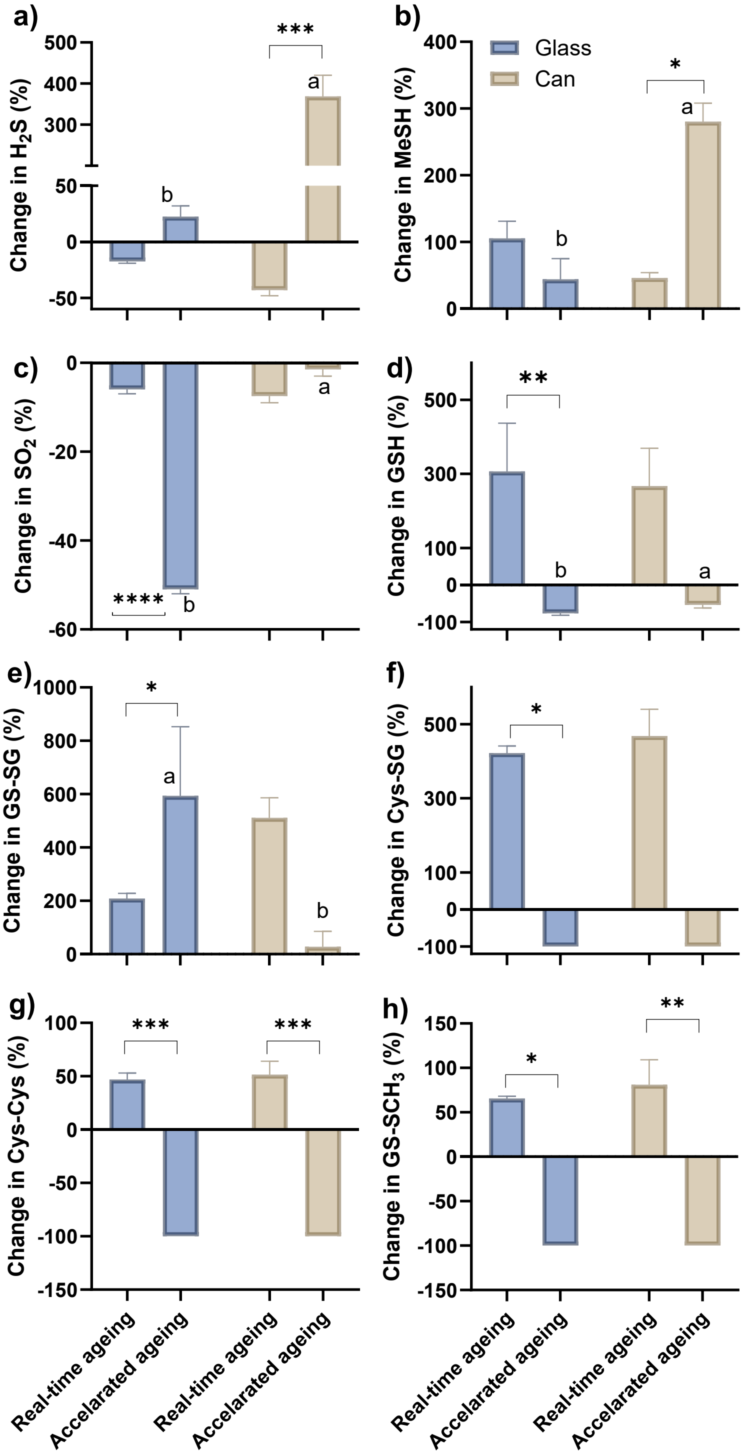

Fig. A.5. Significant percent change of sulfur compounds in Chardonnay under real-time and accelerated ageing conditions, expressed relative to initial levels showing a) H_2S, b) MeSH, c) SO_2, d) GSH, e) GS-SG, f) Cys-SG, g) Cys-Cys, and h) GS-CH_3. Error bars represent the standard error of duplicate measurements. Statistical analyses were performed on raw values (Δ = Tend−T0) whereas the figure displays the corresponding percent changes relative to initial levels. Significant differences were determined by one-way ANOVA followed by Tukey's multiple comparisons test (α = 0.05). Asterisks indicate significant differences between ageing conditions within the same package and different letters above bars denote significant differences between packaging formats under accelerated ageing. Significance levels are represented as follows: *, $P < 0.05$; **, $P < 0.01$; ***, $P < 0.001$; ****, $P < 0.0001$.

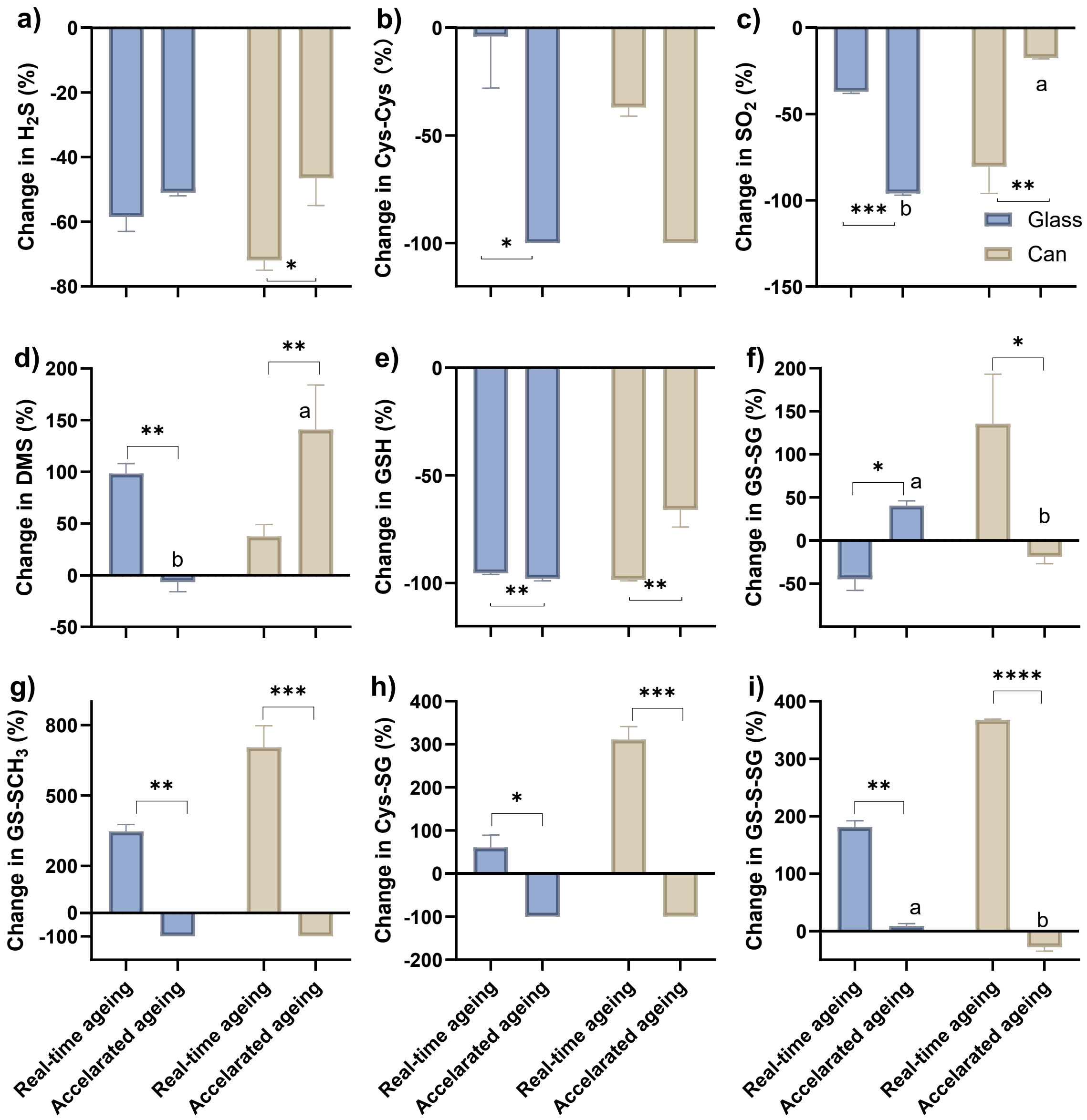

Fig. A.6. Significant changes in percentage of sulfur compounds in Shiraz co-spiked with H_2S/GSH under real-time cellaring (12 months) and accelerated ageing conditions (5 weeks), expressed relative to initial levels. Error bars represent the standard error of duplicate measurements. Panel show a) H_2S, b) Cys-Cys, c) SO_2, d) DMS, e) GSH, f) GS-SG, g) GS-SCH_3, h) Cys-SG, i) GS-S-SG. Statistical analyses were performed on respective raw values ($\Delta = T_{end} - T_0$) whereas the figure displays the corresponding percent changes relative to initial levels. Significant differences were determined by one-way ANOVA followed by Tukey's multiple comparison test ($\alpha = 0.05$). Asterisks indicate significant differences between ageing conditions within the same package, and different letters above bars indicate significant differences between packaging formats under the accelerated ageing. Significance levels are represented as follows: *, $P < 0.05$; **, $P < 0.01$; ***, $P < 0.001$; ****, $P < 0.0001$.

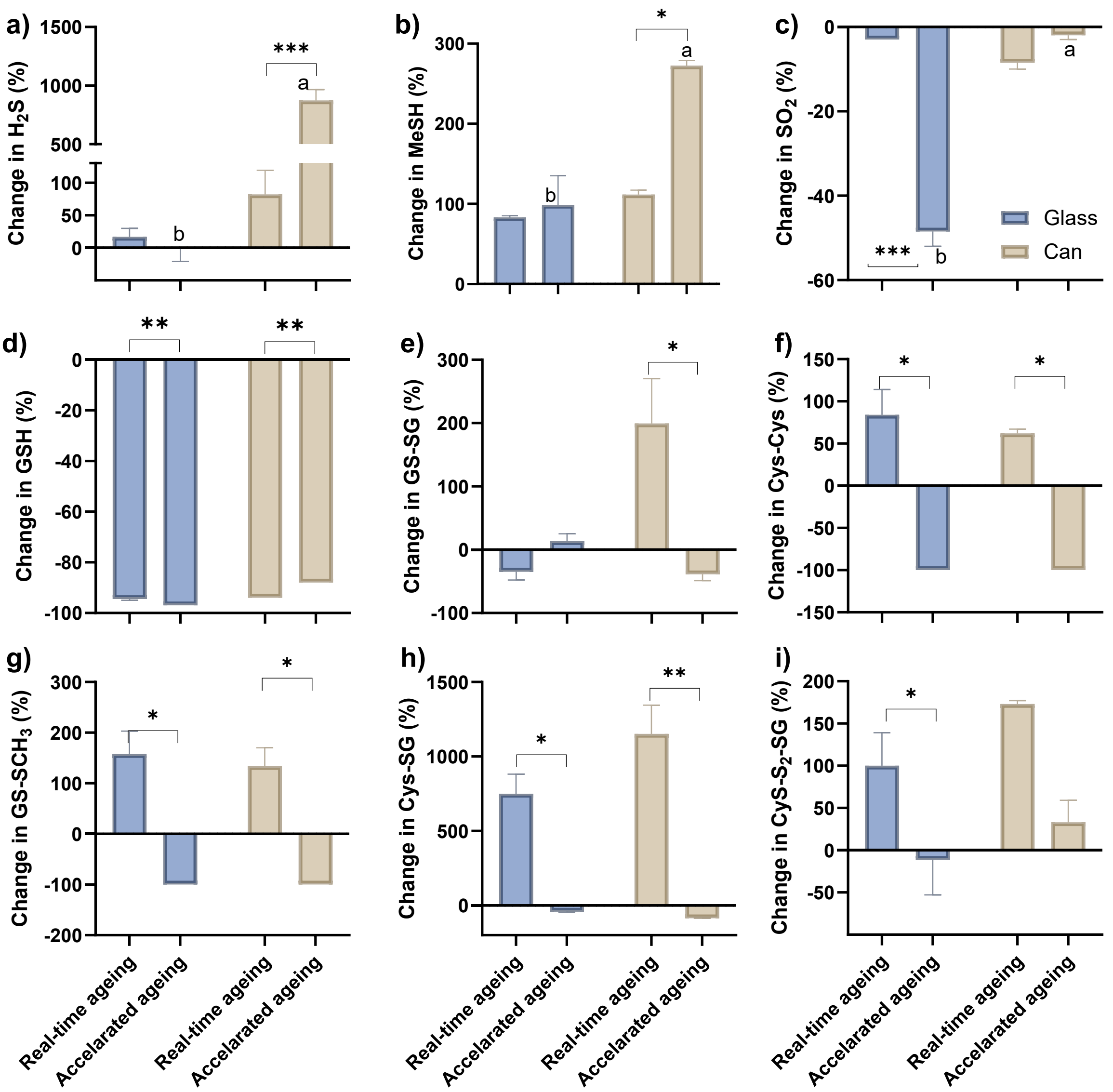

Fig. A.7. Significant changes in percentage of sulfur compounds in Chardonnay co-spiked with H_2S/GSH under real-time cellaring (12 months) and accelerated ageing conditions (5 weeks), expressed relative to initial levels. Error bars represent the standard error of duplicate measurements. Panel show a) H_2S, b) MeSH, c) SO_2, d) GSH, e) GS-SG, f) Cys-Cys, g) $GS\text{-}SCH_3$, h) Cys-SG, i) GS-S-SG. Statistical analyses were performed on raw values ($\Delta = T_{end} - T_0$) whereas the figure displays the corresponding percent changes relative to initial levels. Significant differences were determined by one-way ANOVA followed by Tukey's multiple comparison test ($\alpha = 0.05$). Asterisks indicate significant differences between ageing conditions within the same package, and different letters above bars indicate significant differences between packaging formats under the accelerated ageing. Significance levels are represented as follows: *, $P < 0.05$; **, $P < 0.01$; ***, $P < 0.001$; ****, $P < 0.0001$.

Table A.1. Two-way ANOVA showing the simple effect of ageing time, package, and their interaction, showing P-values of di- and polysulfides in control and co-spiked (GSH/H_2S) Shiraz and Chardonnay wines (based on log_{10}-transformed data) under real-time cellaring.

	Time	Package	Time × Package	Time	Package	Time × Package
		Sh			Sh+	
GS-SG	**<0.0001**	**0.0005**	**0.0003**	**0.0020**	**0.0027**	**0.0150**
Cys-Cys	**0.0035**	0.1787	**0.0058**	**0.0179**	0.2967	0.7929
GS-SCH_3	**0.0003**	**0.0289**	0.6096	**<0.0001**	0.0537	0.6894
Cys-SG	**0.0010**	0.1025	0.3985	**<0.0001**	**0.0303**	**0.0079**
GS-S-SG	**<0.0001**	0.2993	0.2417	**<0.0001**	0.0674	**0.0213**
GS-S_2-SG	**0.0357**	0.6354	0.3287	**0.0022**	0.1764	0.1171
Cys-S_2-SG	**0.0062**	0.8233	0.9987	**<0.0001**	0.4775	0.6867
		Ch			Ch+	
GS-SG	**<0.0001**	**<0.0001**	**<0.0001**	**<0.0001**	**0.0002**	**0.0115**
Cys-Cys	**0.0014**	0.3895	0.6297	**<0.0001**	0.8371	0.9015
GS-SCH_3	**<0.0001**	0.9295	0.4897	**0.0001**	0.8889	0.9765
Cys-SG	**<0.0001**	0.7556	0.5273	**<0.0001**	0.2767	**0.0326**
GS-S-SG	**0.0021**	0.2894	0.1373	**0.0005**	0.6034	0.6495
GS-S_2-SG	**0.0026**	0.8734	0.8438	**0.0020**	0.3602	0.8861
Cys-S_2-SG	**<0.0001**	0.9443	0.1875	**<0.0001**	0.7536	0.8359

Significant P-values ($P < 0.05$) are shown in bold.

Table A.2. Two-way ANOVA showing the simple effect of ageing time, co-spiking treatment (GSH/H_2S), and their interaction, showing P-values of di- and polysulfides in Shiraz and Chardonnay wines (based on $\log_{10}$-transformed data) in different packages under real-time cellaring.

		Time	Treatment	Time × Treatment	Time	Treatment	Time × Treatment
		Sh vs Sh+			Ch vs Ch+		
Can	GS-SG	**0.0007**	**0.0016**	**0.0007**	**0.0241**	**0.0003**	0.3617
	Cys-Cys	**0.0088**	**0.0346**	**0.0235**	**0.0013**	0.9340	0.4611
	GS-SCH$_3$	**0.0020**	**0.0110**	**0.0059**	**0.0157**	0.8883	0.7359
	Cys-SG	**0.0193**	**0.0032**	**0.0005**	**0.0013**	**0.0416**	0.0710
	GS-S-SG	**0.0030**	0.0539	0.1953	0.1020	0.7494	0.8563
	GS-S$_2$-SG	**0.0410**	0.2009	**0.0048**	0.2174	0.7167	0.8968
	Cys-S$_2$-SG	0.0922	0.4059	0.3003	**0.0019**	0.9732	0.5558
Cork	GS-SG	**0.0030**	**0.0010**	**0.0002**	**0.0043**	**0.0002**	**<0.0001**
	Cys-Cys	0.0748	0.1385	0.1151	**0.0339**	0.3947	0.8036
	GS-SCH$_3$	**0.0125**	**0.0064**	**0.0425**	**0.0310**	0.9357	0.9666
	Cys-SG	0.1261	**0.0269**	**0.0158**	**0.0002**	**0.0086**	**0.0045**
	GS-S-SG	**0.0099**	0.1039	0.0903	**0.0094**	0.8109	0.9263
	GS-S$_2$-SG	**0.0053**	0.2421	0.6085	0.1737	0.5039	0.7221
	Cys-S$_2$-SG	**0.0303**	0.5659	0.9738	**0.0011**	0.3835	0.6169
SC1	GS-SG	**0.0299**	0.0003	0.0002	0.0012	0.0002	<0.0001
	Cys-Cys	0.1745	0.4852	0.4262	0.0640	0.8454	0.4246
	GS-SCH$_3$	**0.0192**	0.0240	0.0026	0.0382	0.8870	0.1047
	Cys-SG	0.1140	0.0134	0.0332	0.0110	0.0883	0.3955
	GS-S-SG	**0.0008**	0.0002	0.0013	0.0007	0.5165	0.3592
	GS-S$_2$-SG	0.1911	0.7298	0.9590	0.0875	0.0837	0.1771
	Cys-S$_2$-SG	**0.0442**	0.8634	0.8643	0.0152	0.8997	0.3673
SC2	GS-SG	**0.0306**	**0.0001**	**0.0005**	**<0.0001**	**<0.0001**	**<0.0001**
	Cys-Cys	0.1475	0.3348	0.1793	0.0977	0.4606	0.2580
	GS-SCH$_3$	**0.0059**	**0.0198**	**0.0443**	0.1006	0.4502	0.2974
	Cys-SG	0.2655	**0.0134**	0.0755	**0.0241**	**0.0015**	0.2808
	GS-S-SG	**0.0299**	0.6077	0.7319	0.0006	0.3854	0.0472
	GS-S$_2$-SG	0.2355	0.6835	0.6721	0.1559	0.5515	0.4291
	Cys-S$_2$-SG	0.0807	0.0979	0.8443	**0.0115**	0.9168	0.0961
SC3	GS-SG	0.0786	**0.0066**	**0.0019**	**0.0008**	**0.0009**	**<0.0001**
	Cys-Cys	0.1011	0.4139	0.9939	**0.018**	0.6750	**0.0884**
	GS-SCH$_3$	**0.0072**	**0.0252**	0.0792	**0.0124**	0.7700	0.7535
	Cys-SG	0.0859	**0.0074**	**0.0403**	**0.0026**	0.1321	0.2905
	GS-S-SG	**0.0099**	0.8223	0.0531	**0.0012**	0.3628	0.1104
	GS-S$_2$-SG	0.2496	0.2714	0.4415	**0.0440**	0.2182	0.5491
	Cys-S$_2$-SG	0.0641	0.8159	0.6584	**0.0041**	0.5303	0.0764

Significant P-values ($P < 0.05$) are shown in bold.

Table A.3. Concentrations of brine-releasable methanethiol (MeSH) and dimethyl sulfide (DMS) in control and co-spiked (GSH/H_2S) Shiraz and Chardonnay wines monitored during real-time cellaring. Values are expressed as mean ± SE (n = 2) at 3, 6, and 12 months, whereas initial concentrations of MeSH and DMS were determined without replication.

		MeSH(µg/L)				DMS(µg/L)			
		0 m	3 m	6 m	12 m	0 m	3 m	6 m	12 m
Sh	Can	2.9	2.4 ± 0.2	1.7 ± 0.1	3.6 ± 1.6	31.8	38.1 ± 9.31	44.0 ± 14.1	47.3 ± 3.3
	Cork	2.6	2.3 ± 0.1	2.3 ± 0.1	2.3 ± 0.1	30.0	27.4 ± 1.75	41.3 ± 7.5	45.5 ± 3.7
	SC1	2.6	2.3 ± 0.0	2.3 ± 0.4	2.8 ± 0.5	30.0	29.0 ± 0.45	53.2 ± 19.0	50.8 ± 6.1
	SC2	2.6	2.7 ± 0.5	1.3 ± 1.8	2.3 ± 0.5	30.0	42.9 ± 18.9	39.9 ± 6.8	45.9 ± 2.3
	SC3	2.6	2.6 ± 0.3	1.9 ± 0.3	3.8 ± 1.6	30.0	34.3 ± 8.0	43.5 ± 11.7	50.6 ± 2.0
Sh+	Can	2.9	2.6 ± 0.4	2.1 ± 0.4	2.1 ± 0.2	31.8	37.2 ± 14.0	37.2 ± 5.6	43.8 ± 5.0
	Cork	2.6a	3.1 ± 1.0ab	2.2 ± 0.0b	2.6 ± 0.4ab	30.0ab	38.2 ± 15.0ab	41.7 ± 2.8b	52.3 ± 2.3a
	SC1	2.6	2.6 ± 0.2	1.9 ± 0.4	2.2 ± 0.2	30.0	29.2 ± 1.74	39.3 ± 3.3	59.5 ± 4.0
	SC2	2.6a	2.5 ± 0.0ab	2.1 ± 0.0b	2.9 ± 1.1ab	30.0	32.5 ± 6.9	40.0 ± 1.2	59.5 ± 16.0
	SC3	2.6	2.4 ± 0.1	2.2 ± 0.1	3.0 ± 1.0	30.0	29.7 ± 3.4	40.1 ± 7.8	59.8 ± 11.2
Ch	Can	8.2a	2.3 ± 0.1ab	2.4 ± 0.0b	4.1 ± 0.3ab	70.4b	76.2 ± 24.6ab	81.0 ± 0.2a	105.7 ± 15.0ab
	Cork	8.2	2.7 ± 0.6	2.9 ± 0.3	5.0 ± 0.4	70.4	77.5 ± 28.9	92.2 ± 4.1	142.3 ± 11.6
	SC1	8.2	2.9 ± 0.2	4.0 ± 0.5	5.7 ± 1.0	70.4b	62.4 ± 6.5ab	94.4 ± 0.4a	129.6 ± 23.2ab
	SC2	8.2	4.5 ± 1.8	4.2 ± 0.9	7.3 ± 1.1	70.4b	77.8 ± 28.3ab	81.5 ± 0.4a	123.2 ± 15.5ab
	SC3	8.2a	2.4 ± 0.0b	4.7 ± 0.3ab	8.9 ± 1.1ab	70.4a	54.8 ± 0.0b	87.6 ± 7.1ab	141.5 ± 23.3ab
Ch+	Can	2.5ab	2.3 ± 0.2ab	3.2 ± 0.3b	5.2 ± 0.2a	55.9	65.1 ± 23.9	93.4 ± 16.0	102.1 ± 7.4
	Cork	2.8	2.4 ± 0.1	3.0 ± 0.4	4.7 ± 1.3	54.2b	55.0 ± 0.1a	75.4 ± 7.5ab	115.2 ± 28.8ab
	SC1	2.8c	2.4 ± 0.0b	3.0 ± 0.1abc	5.1 ± 0.1a	54.2b	81.1 ± 33.3ab	79.0 ± 0.9a	113.9 ± 17.2ab
	SC2	2.8a	2.4 ± 0.0b	2.7 ± 0.2ab	6.0 ± 0.4ab	54.2b	64.3 ± 0.0a	76.9 ± 4.6ab	124.0 ± 10.6ab
	SC3	2.8b	2.4 ± 0.0b	3.0 ± 0.0a	6.6 ± 1.0ab	54.2	61.8 ± 4.0	82.8 ± 10.7	121.8 ± 19.0

Different lowercase letters designate significant differences over 12 months within the same package format (from two-way repeated measures ANOVA followed by Tukey's multiple comparison test, $\alpha = 0.05$).

Table A.4. Two-way ANOVA showing the simple effect of ageing time, package, and their interaction, showing P-values of three VSCs (H_2S, MeSH, DMS), SO_2, and GSH in control and co-spiked (GSH/H_2S) Shiraz and Chardonnay under real-time cellaring.

	Time	Package	Time × Package	Time	Package	Time × Package
		Sh			Sh+	
H_2S	**0.0008**	0.2889	0.7406	**<0.0001**	0.0577	0.6376
MeSH	0.0867	0.2883	0.7628	**0.0731**	0.6725	0.6965
DMS	**0.0191**	0.7195	0.8474	**0.0003**	0.8828	0.6511
SO_2	**<0.0001**	**0.0004**	**0.0047**	**<0.0001**	**0.0001**	**0.0017**
GSH	**<0.0001**	0.1342	0.8195	**<0.0001**	0.1780	0.1019
		Ch			Ch+	
H_2S	**0.0001**	0.0811	**0.0005**	**0.0198**	**0.0041**	**0.0017**
MeSH	**<0.0001**	**0.0422**	**0.0003**	**<0.0001**	0.1618	0.1209
DMS	**<0.0001**	0.4326	0.5759	**0.0001**	0.8021	0.8021
SO_2	**<0.0001**	0.2392	0.9907	**<0.0001**	0.6681	0.9067
GSH	**0.0097**	0.8389	0.8785	**<0.0001**	0.8759	0.9997

Significant P-values (P < 0.05) are shown in bold.

Table A.5. One-way ANOVA showing P-values for analytes with at least one packaging format showing a significant difference between control vs co-spiked (GSH/H_2S) treatments in Shiraz and Chardonnay under accelerated ageing.

	Can	Ampoule
	Sh vs Sh+	
GSH	0.1227	**0.0012**
GS-SG	0.3704	**0.0286**
Cys-Cys	**0.0375**	0.9704
Cys-SG	**0.0376**	0.0767
GS-S-SG	**0.0371**	**0.0298**
GS-S_2-SG	**0.0029**	0.9509
	Ch vs Ch+	
H_2S	**0.0001**	0.8522
MeSH	0.9876	**0.0093**
GSH	**0.0002**	**0.0002**

Significant P-values (P < 0.05) are shown in bold.

Table A.6. One-way ANOVA results showing the effect of N_2, SO_2, SO_2 + AA, or Cu addition on change (absolute and relative) compared to initial levels for sulfur compounds in co-spiked (H_2S/GSH) Shiraz and Chardonnay wines under accelerated ageing. Only analytes with significant differences are included.

	Untreated (μg/L)	% Change	Treated (μg/L)	% Change	P-value
N_2			Sh+		
H_2S	−1.25	−51%	0.474	39%	0.0109
SO_2	−54.6	−96%	−33.4	−59%	<0.0001
GS-SCH_3	−9.89	−100%	270	651%	0.0002
Cys-SG	−14.0	−100%	−1.00	−8%	0.0035
N_2			Ch+		
H_2S	−0.013	−1%	7.97	515%	0.0253
MeSH	2.34	98%	5.35	338%	0.0132
SO_2	−69.8	−48%	−29.0	−20%	0.0042
Cys-Cys	−1.84	−100%	1.36	76%	<0.0001
GS-SCH_3	−2.04	−100%	−0.088	−5%	<0.0001
Cys-SG	5.68	−41%	42.8	377%	0.0004
SO_2			Sh+		
SO_2	−54.6	−96%	−70.9	−96%	0.0001
SO_2 + AA			Sh+		
SO_2	−54.6	−96%	−71.2	−97%	0.0001
SO_2 + AA			Ch+		
SO_2	−69.8	−48%	−103	−65%	0.0160
Cu			Sh+		
GS-SG	2980	40%	4060	211%	0.0127
Cys-SG	−14.0	−100%	31.4	224%	<0.0001
Cu			Ch+		
MeSH	2.34	98%	−0.512	−22%	0.0423
SO_2	−69.8	−48%	−143	−98%	<0.0001
GS-S-SG	1.36	0.4%	11.0	354%	0.0026
